# CDACHIE: chromatin domain annotation by integrating chromatin interaction and epigenomic data with contrastive learning

**DOI:** 10.1093/bioinformatics/btaf464

**Published:** 2025-08-22

**Authors:** Asato Yoshinaga, Osamu Maruyama

**Affiliations:** Graduate School of Design, Kyushu University, Fukuoka, 815-8540, Japan; Faculty of Design, Kyushu University, Fukuoka, 815-8540, Japan

## Abstract

**Motivation:**

Chromatin domain annotation identifies functional genomic regions, such as active and inactive zones, based on epigenomic features like histone modifications, DNA methylation, and chromatin accessibility. While recent methods have utilized both chromatin interaction data (e.g. Hi-C) and epigenomic data, they often overlook the direct relationship between these data types.

**Results:**

In this study, we introduce Chromatin Domain Annotation using Contrastive Learning for Hi-C and Epigenomic Data (CDACHIE), a method for identifying chromatin domains from Hi-C and epigenomic data. Our approach leverages contrastive learning to generate aligned representative vectors for both data types at each genomic bin. The concatenated vectors are then clustered using *K*-means to classify distinct chromatin domain types. CDACHIE achieves superior performance in Variance Explained, evaluated across gene expression, replication timing, and ChIA-PET data. This highlights its robust ability to integrate semantic associations between Hi-C and epigenomic features within the embedding space.

**Availability and implementation:**

The source code is available at GitHub: https://github.com/maruyama-lab-design/CDACHIE. An archival snapshot of the code used in this study is available on Zenodo: https://doi.org/10.5281/zenodo.15751780.

## 1 Background

Chromatin-domain annotation seeks to partition the genome into regions that share regulatory properties, thereby providing a concise map of functional organization (Dixon *et al.* 2012, Rao *et al.* 2014, Libbrecht *et al.* 2021). Early epigenome-centric methods such as ChromHMM (Ernst and Kellis 2012) and Segway (Hoffman *et al.* 2012) successfully captured 1D chromatin states from histone-modification and accessibility profiles, but were developed before genome-wide Hi-C data became widely available and therefore did not incorporate information on the emerging 3D chromatin architecture.

The advent of Hi-C uncovered hierarchical 3D structures—A/B compartments, TADs, and loops—that are tightly coupled to transcriptional activity (Lieberman-Aiden *et al.* 2009, Rao *et al.* 2014). To exploit this extra dimension, hybrid algorithms have emerged. Segway-GBR (Libbrecht *et al.* 2015) and SPIN (Wang *et al.* 2021) treat Hi-C contacts as graph-based regularizers for epigenome-driven models, whereas SCI derives domains from Hi-C alone (Ashoor *et al.* 2020). The current state-of-the-art integrative method, HMM_combined (Shokraneh *et al.* 2023), concatenates an epigenomic vector with a LINE-derived Hi-C embedding before learning a multi-state hidden Markov model. Because these approaches fuse modalities only at the decision level or treat them as independent features, important correspondences between structure and function may be lost.

We therefore introduce CDACHIE, a contrastive-learning framework that explicitly aligns Hi-C and epigenomic representations at the level of individual genomic bins. Two modality-specific encoders—one for the multi-track epigenomic signals and the other for Hi-C contact profiles—are jointly trained to maximize the agreement between structural and functional embeddings; the resulting vectors are concatenated and clustered to yield domain labels. By unifying complementary assays in a shared latent space, CDACHIE delivers more nuanced and biologically coherent annotations, as demonstrated below.

## 2 Materials and methods

**Figure 1.**
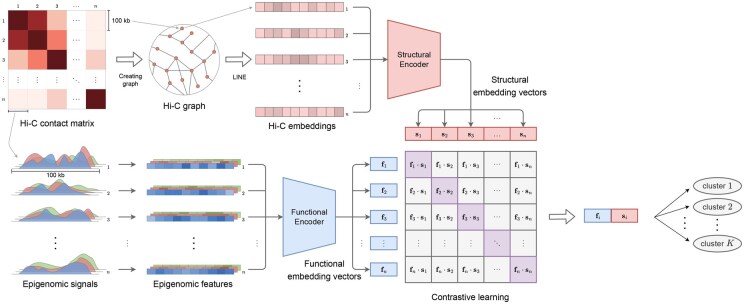
Overview of CDACHIE. Each chromosome is partitioned into non-overlapping 100 kb bins. For every bin, the corresponding Hi-C contact profile and multi-track epigenomic signals serve as inputs to two modality-specific neural networks—a structural encoder (Hi-C) and a functional encoder (epigenome). Both encoders map their inputs to *d*-dimensional embeddings, denoted $s_{i},f_{i}\in R^{d}$. The two embeddings from the same bin are concatenated into a 2*d*-dimensional vector, which is then clustered into *K* groups by *K*-means; each cluster is interpreted as a distinct chromatin-domain type.

Figure 1 illustrates an overview of the proposed method, CDACHIE. First, each chromosome sequence is divided into bins with a width of 100 kb. CDACHIE operates within a contrastive learning framework to generate embedding vectors derived from Hi-C and epigenomic data for each bin, enabling the identification of chromatin domain types. An input instance to CDACHIE comprises a pair of a Hi-C embedding vector generated using the LINE algorithm and an epigenomic feature vector derived from the same bin. These vectors are processed using two neural networks: a structural encoder and a functional encoder. The structural encoder transforms the Hi-C embedding vector, whereas the functional encoder processes the epigenomic feature vector. Both encoders output embedding vectors with identical dimensionalities. These outputs are referred to as the structural embedding vector and functional embedding vector, respectively. The structural and functional embedding vectors of each bin are concatenated and subsequently grouped into *K* clusters using *K*-means clustering. Each cluster corresponds to a distinct chromatin domain type.

### 2.1 Datasets

We used publicly available Hi-C maps for the GM12878 and K562 cell lines (GSE63525), along with 12 epigenomic tracks and evaluation data including gene expression, replication timing, and CTCF and RNAP II ChIA-PET data. Details of the data sources and preprocessing procedures are provided in Supplementary Materials 1 and 2, available as supplementary data at *Bioinformatics* online, and Table 1, available as supplementary data at *Bioinformatics* online.

### 2.2 Chromatin domain identifier CDACHIE

Figure 1 provides an overview of CDACHIE’s architecture, which consists of (i) a structural encoder for Hi-C data and (ii) a functional encoder for epigenomic signals. Initially, Hi-C contact matrices are transformed into embeddings using the node embedding method LINE (see Supplementary Material 3, available as supplementary data at *Bioinformatics* online). These embeddings serve as input to the structural encoder. The outputs of the encoders are referred to as structural and functional embeddings, respectively. Layer configurations and hyperparameters are summarized in Supplementary Material 4, available as supplementary data at *Bioinformatics* online. Both encoders are optimized using a bidirectional InfoNCE loss that increases the cosine similarity of matching bins and decreases it for non-matching pairs (Oord *et al.* 2018) (see Supplementary Material 5, available as supplementary data at *Bioinformatics* online).

The structural and functional embeddings from the same bin are concatenated and clustered into *K* groups using *K*-means. We report Variance Explained (VE) and the Observed/Expected (O/E) ratio; formal definitions are provided in Supplementary Material 6, available as supplementary data at *Bioinformatics* online.

## 3 Results

The domain annotations generated by CDACHIE were compared to those generated by HMM_combined and GMM_GBR. The number of CDACHIE clusters was set to be six, which is the same as those of HMM_combined, GMM_GBR, and the Hi-C subcompartments. We have also executed additional experiments with the value of *K* in the range from 7 to 10 (see Supplementary Material 7, available as supplementary data at *Bioinformatics* online). According to the increase of *K*, the interpretability decreases, as also mentioned in Shokraneh *et al.* (2023).

The two existing methods are described as follows: HMM_combined is a state-of-the-art method for domain annotation using both epigenomic and Hi-C data (Shokraneh *et al.* 2023). It is modeled by a hidden Markov model (HMM) whose input is the concatenation of the 12 epigenomic features mentioned above and an 8D Hi-C embedding vector generated using LINE. GMM_GBR, a method that applies graph-based regularization (GBR) to a Gaussian mixture model (GMM), was referred to as such in Shokraneh *et al.* (2023), while the original formulation of GBR for domain annotation was introduced in Libbrecht *et al.* (2015) as part of Segway-GBR. GMM_GBR, introduced by Shokraneh *et al.* (2023), serves as a baseline GMM model. Due to the lack of available annotation files and the fact that the published code could not be executed in our environment, the GMM_GBR results used in this study were limited to those explicitly reported in the original paper (Shokraneh *et al.* 2023). In contrast, we ran the HMM_combined method ourselves and obtained annotations for a broader set of conditions and cell types.

**Figure 2.**
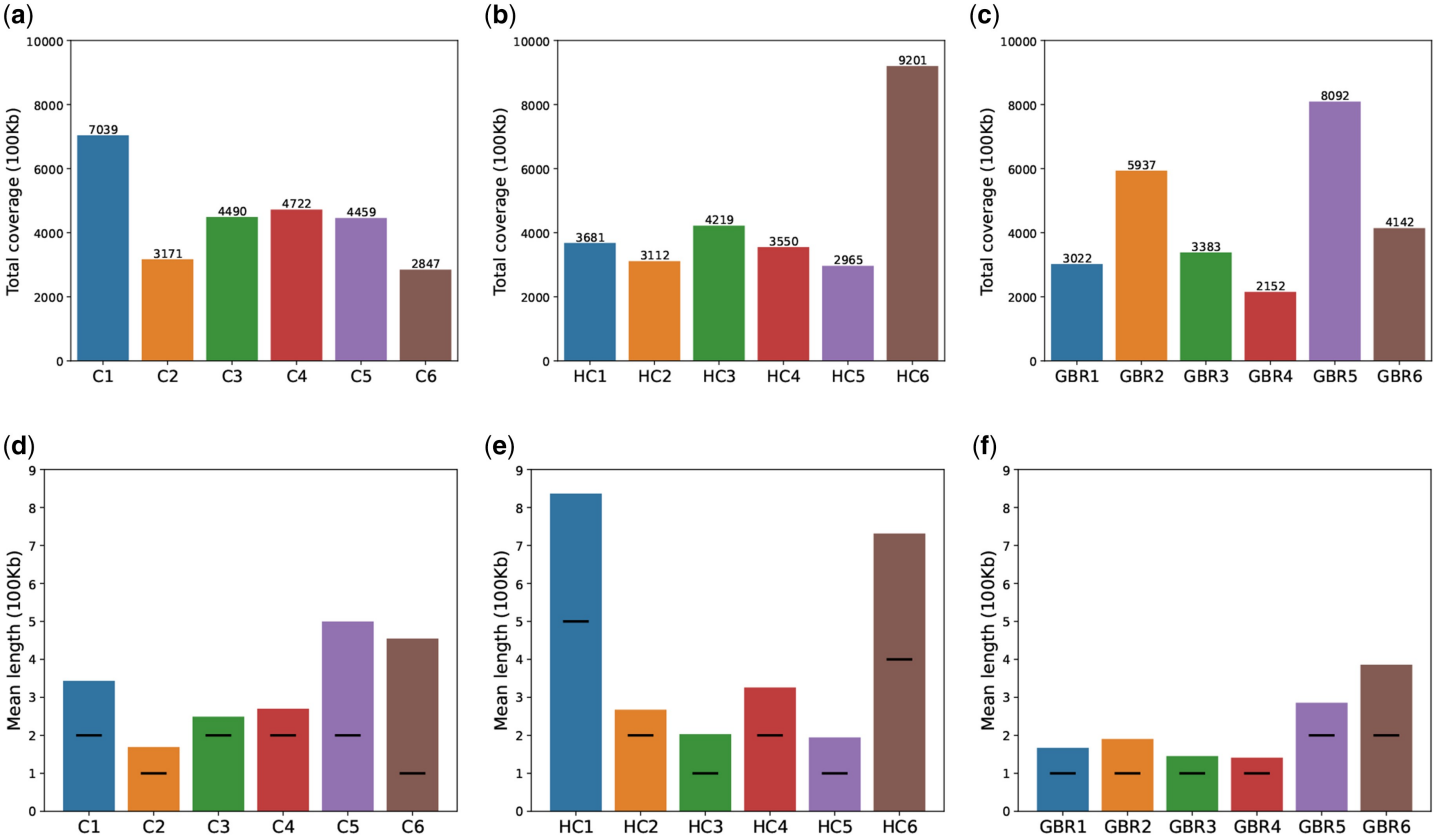
(a), (b), and (c) display the number of bins for each domain type (domain coverage) in the domain annotations of CDACHIE, HMM_combined, and GMM_GBR, respectively. (d), (e), and (f) present the mean lengths of contiguous bins with the same domain type of CDACHIE, HMM_combined, and GMM_GBR, respectively. The horizontal lines within the bars represent the median.

As foundational information for the analysis on the GM12878 cell line data, we present Fig. 2a–c, which illustrate the number of bins for each domain type of CDACHIE, HMM_combined and GMM_GBR, respectively, and (d), (e), and (f), which display the mean length of contiguous bins with the same domain type. The result for the K562 cell line data using CDACHIE and HMM_combined is shown in Fig. 8, available as supplementary data at *Bioinformatics* online.

### 3.1 CDACHIE outperforms alternative methods

We compared the domain annotations generated by CDACHIE, HMM_combined, and GMM_GBR using four evaluation metrics: variance explained for gene expression (GE VE), variance explained for replication timing (RT VE), observed-to-expected ratio for CTCF ChIA-PET loops (CTCF O/E), and observed-to-expected ratio for RNAP II ChIA-PET loops (RNAP II O/E).

**Figure 3.**
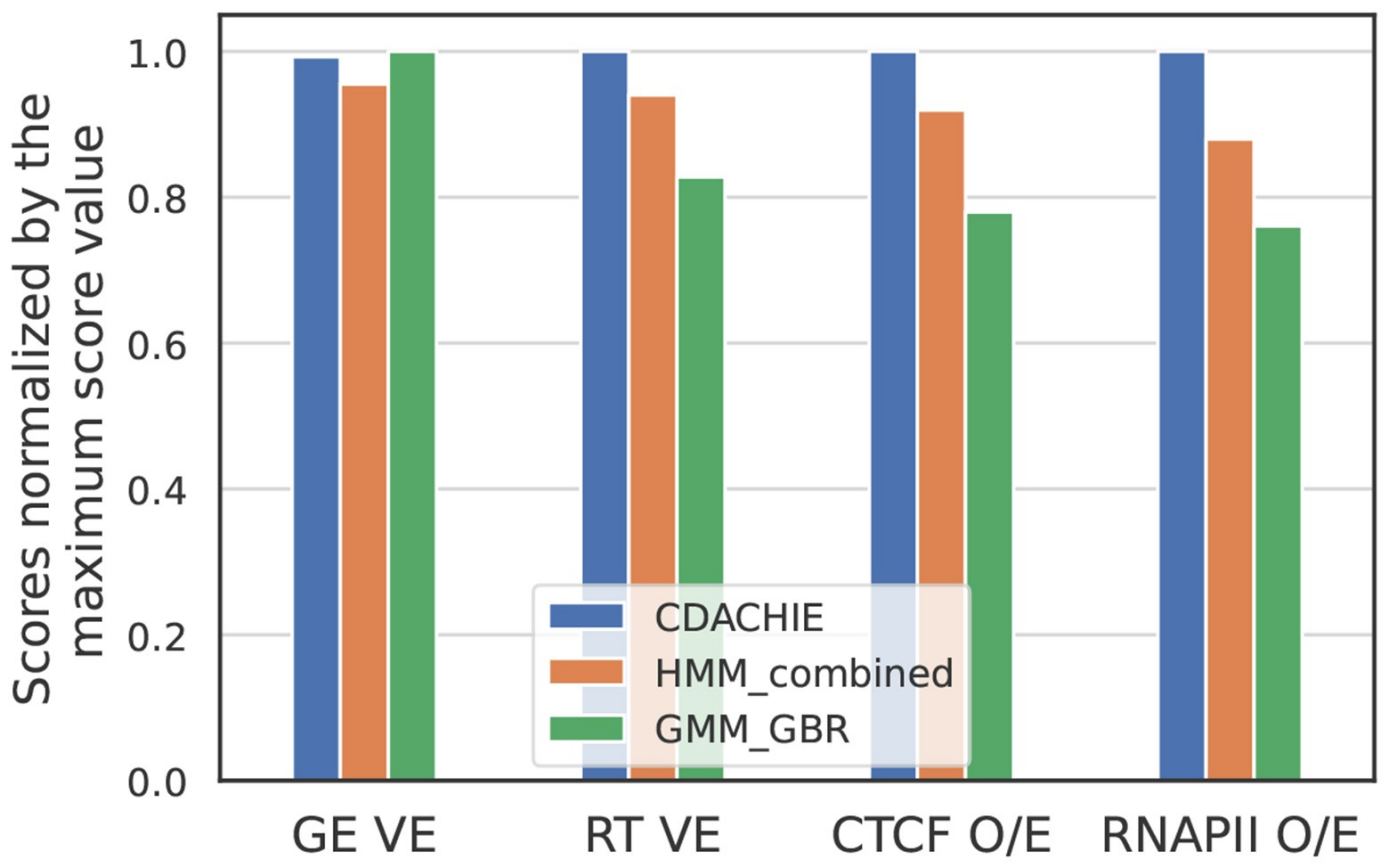
Scores of GE VE, RT VE, CTCF O/E, and RNAP II O/E of the domain annotations generated by CDACHIE, HMM_combined, and GMM_GBR. The *y*-axis shows the scores normalized by the maximum score of each metric.

Figure 3 shows that while CDACHIE achieves a slightly lower GE VE score than that of GMM_GBR, it significantly outperforms both HMM_combined and GMM_GBR in terms of RT VE, CTCF O/E, and RNAP II O/E. This result demonstrates the ability of CDACHIE to generate more distinctive domain annotations across various functional and structural features. Analysis of the K562 cell line data (Fig. 9, available as supplementary data at *Bioinformatics* online) also demonstrates the superior performance of CDACHIE over HMM_combined in terms of RT VE and CTCF O/E scores, underscoring its robustness across different cell lines.

**Figure 4.**
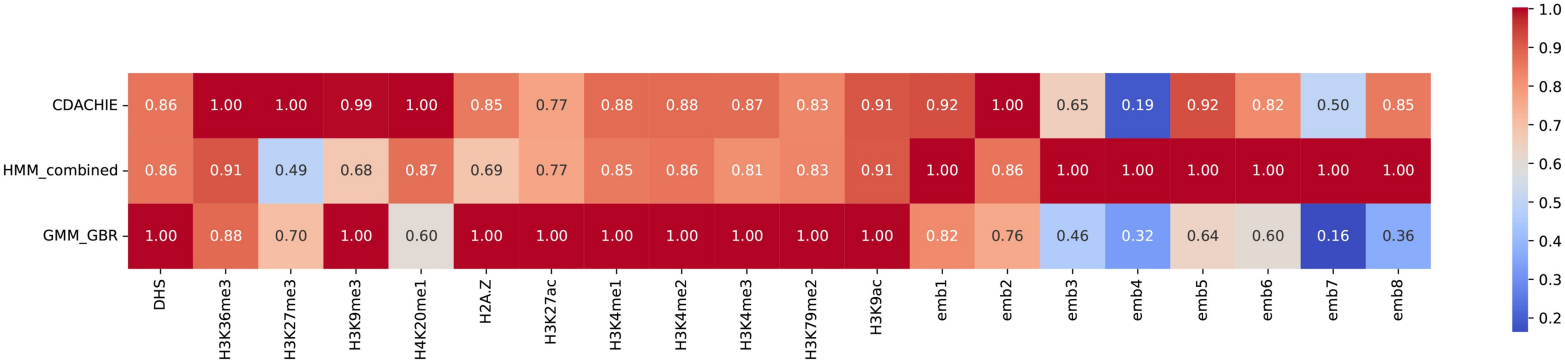
The normalized proportion of variance explained (VE) for each input signal in the domain annotations of CDACHIE, HMM_combined, and GMM_GBR. Each VE is normalized by dividing by the maximum VE across the three annotations, representing the proportions for 12 epigenomic signals and each component of the 8D Hi-C embedding vectors generated using LINE. The dimensionality of the Hi-C embedding vector is set to eight to match the HMM_combined.

Figure 4 shows the normalized VE for input signals. This analysis reveals the contribution of each input signal to the domain annotations. HMM_combined relies heavily on Hi-C embeddings, whereas GMM_GBR emphasizes epigenomic signals. CDACHIE achieves a balanced contribution from both input signals, reflecting its superior integration strategy. Analysis of K562 cell line data (Fig. 10, available as supplementary data at *Bioinformatics* online) also demonstrates the balanced contribution of CDACHIE to the integration of epigenomic and Hi-C data, further validating its robustness across cell lines.

Figures 3 and 4 implies that distinctive results among CDACHIE, HMM_combined, and GMM_GBR can be attributed to their respective approaches toward integrating epigenomic signals and Hi-C data. The strategy of CDACHIE to effectively integrate these two data sources seems to be the key to its superior performance.

### 3.2 CDACHIE identifies distinct domain types

**Figure 5.**
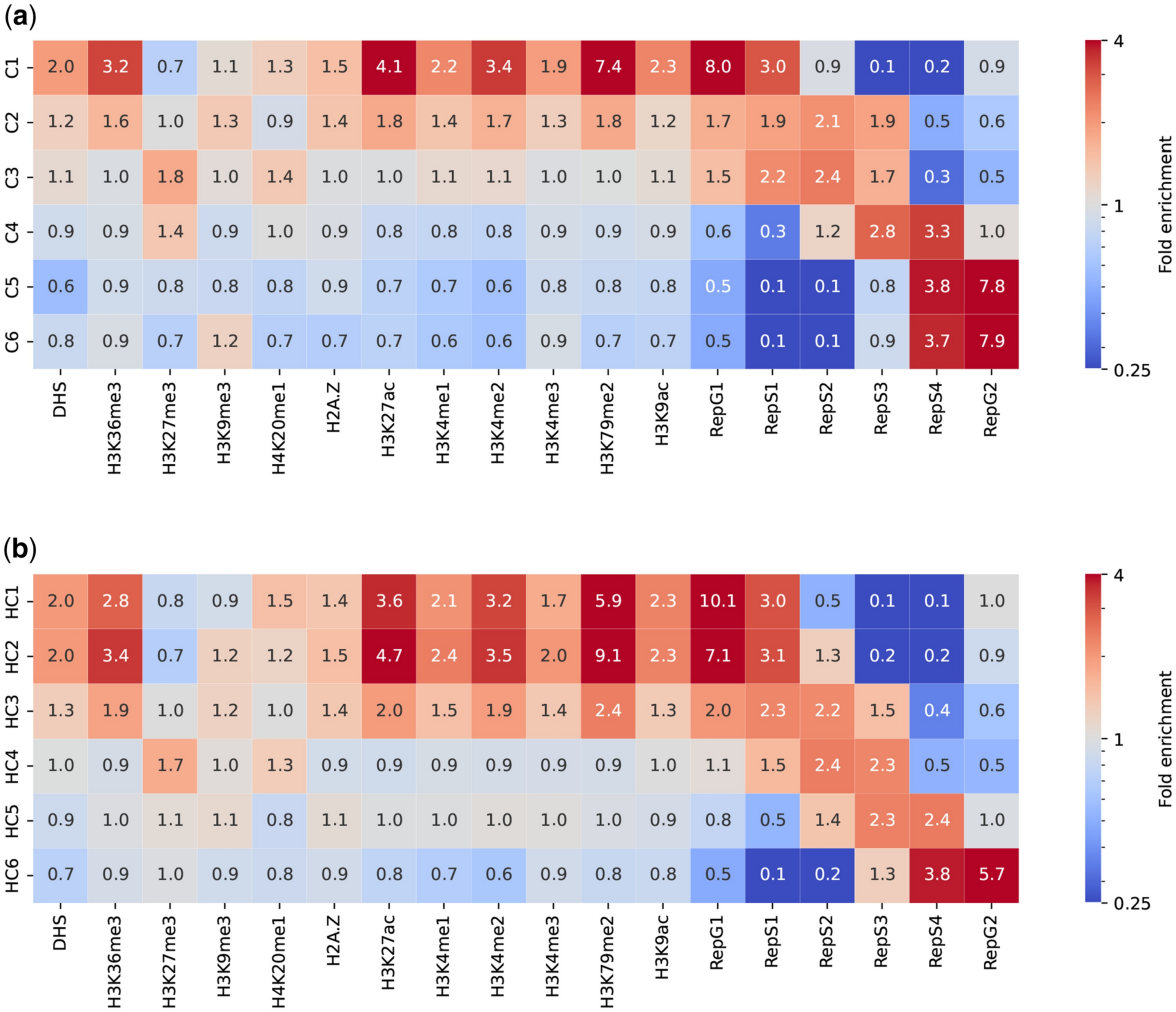
Fold enrichment of epigenomic markers and replication timing signals for each domain type identified using CDACHIE (a) and HMM_combined (b) domain annotations. Fold enrichment is calculated as the ratio of the median signal value within a domain type to the median value across all genomic bins.

Figure 5 depicts the fold enrichment of epigenomic markers and the replication timing signals for each domain type. For CDACHIE, the results (Fig. 5a) show that C1 and C2 exhibited the highest and second-highest fold enrichments across the most active histone modifications, while C3 demonstrates neutral enrichment, and C4-C6 show lower fold enrichments.

**Figure 6.**
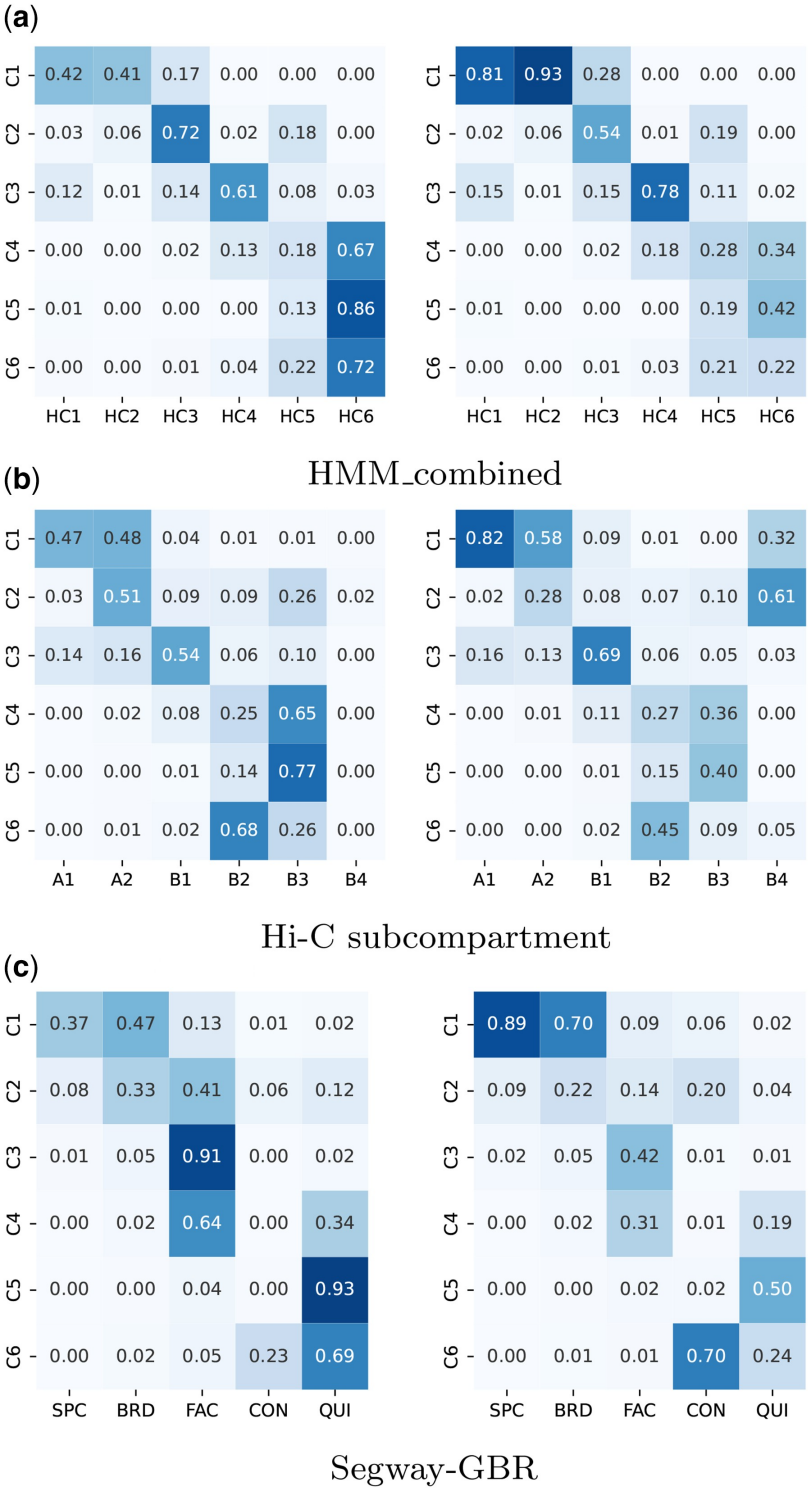
Overlap rates between the domain types identified by CDACHIE and (a) those derived from HMM_combined, (b) Hi-C subcompartments, and (c) those from Segway-GBR. The left panels show row-normalized values, while the right panels show column-normalized values, respectively.

Figure 5b shows the corresponding results for HMM_combined, where active histone modifications are broadly enriched in HC1 and HC2. The remaining domain types exhibit lower enrichment in that order. These patterns arise from the overlapping relationships shown in Fig. 6a. Specifically, C1 encompasses the combination of HC1 and HC2, C2 and C3 align with HC3 and HC4, respectively, and C4-C6 primarily span HC6 and HC5. This suggests that the active domain types identified by CDACHIE are more distinct than those identified by HMM_combined. The domain annotations of CDACHIE and HMM_combined showed a similarity of 0.33 in the Adjusted Rand Index (ARI) (see Supplementary Material 8, available as supplementary data at *Bioinformatics* online).

Figure 11, available as supplementary data at *Bioinformatics* online shows that the fold enrichment analysis for the K562 cell line is consistent with the observed overlap relationships. Figure 12, available as supplementary data at *Bioinformatics* online illustrates the overlaps between the domain types identified by CDACHIE and HMM_combined in the K562 cell line, indicating that C1 corresponds to HC1, C2 to HC2, C3 to HC4, C4 and C5 to HC5, and C6 to HC6. The ARI between the domain types identified by CDACHIE and HMM_combined in the K562 cell line was 0.54, which is higher than that observed in the GM12878 cell line.

We further visualized the distribution of histone modifications over a 2D UMAP projection of the concatenated structural and functional embeddings (see Supplementary Material 9, available as supplementary data at *Bioinformatics* online and Fig. 3, available as supplementary data at *Bioinformatics* online). This visualization shows that the CDACHIE embeddings form distinct clusters corresponding to H3K27ac-enriched (active; C1-2) and H3K27me3-enriched (repressive; C3-4) regions. The projections of the remaining histone modifications are given in Fig. 4, available as supplementary data at *Bioinformatics* online.

In addition, we evaluated dinucleotide distribution patterns across chromatin domain types CpG is highly enriched in domain types C1 and C3 (Supplementary Material 10, available as supplementary data at *Bioinformatics* online, Fig. 5, available as supplementary data at *Bioinformatics* online). Other GC-rich dinucleotides show similar trends, supporting active-inactive domain boundaries.

### 3.3 Functional characteristics of domain types

**Figure 7.**
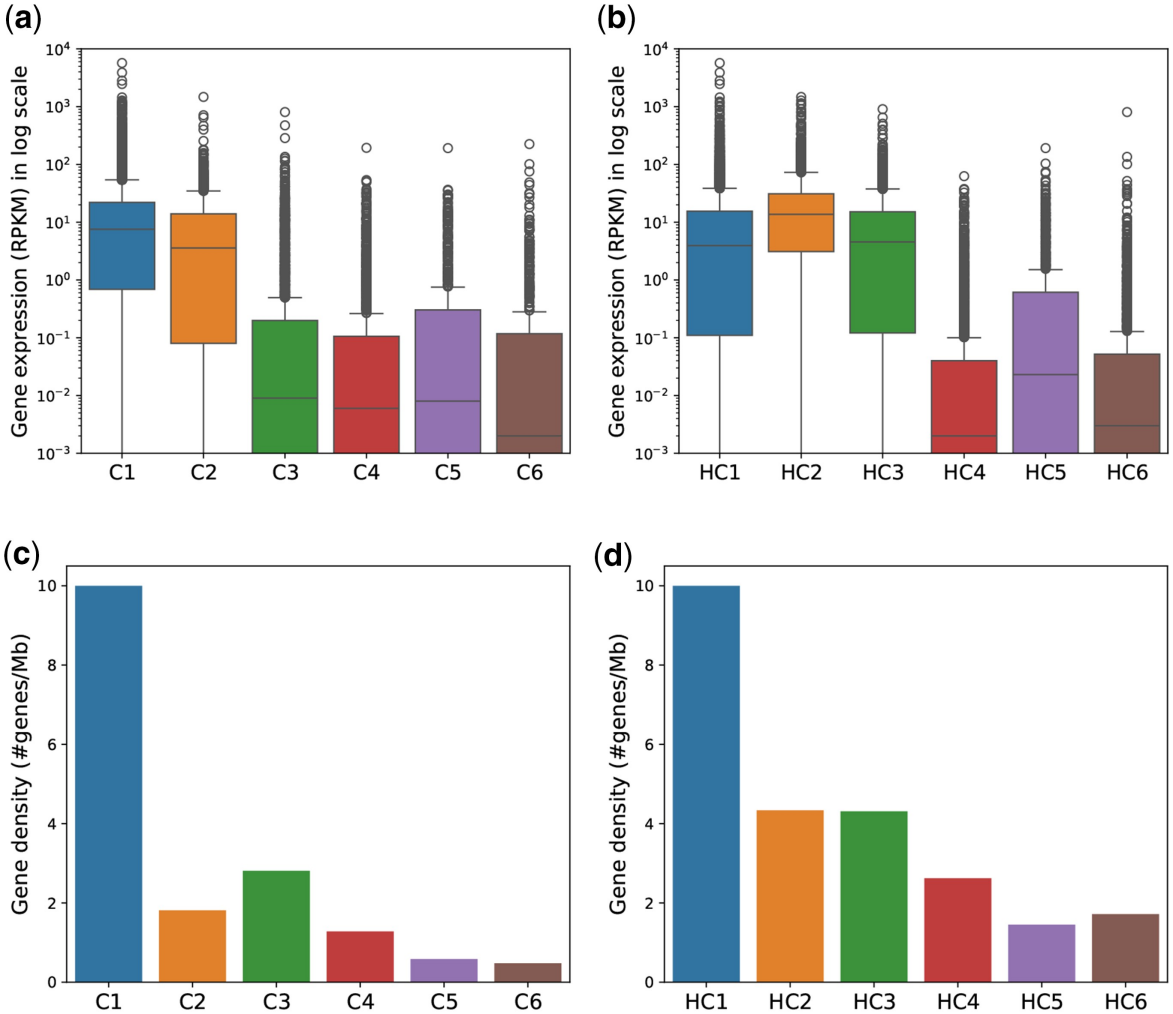
Distribution of gene expression values in RPKM for each domain type of CDACHIE (a) and HMM_combined (b) domain annotations. Gene density corresponding to each domain type of CDACHIE (c) and HMM_combined (d) domain annotations.

Figure 7 shows that CDACHIE successfully identified two distinct types of domains: C1 and C2. C1 exhibits both high gene expression and gene density, whereas C2 shows high gene expression but lower gene density. This finding suggests that CDACHIE can distinguish between regions based on gene expression and density.

In contrast, the active domain types of HMM_combined are HC1, HC2, and HC3 (Fig. 7b). Although the gene density of HC1 is the highest, the gene densities of HC2 and HC3, which are relatively high, are almost the same. Thus, there is no clear distinction between HC2 and HC3 in terms of the gene expression and density.

Consistent with the observations in GM12878, our analysis of the K562 cell type (see Fig. 13, available as supplementary data at *Bioinformatics* online) revealed a similar grouping pattern of active and inactive domains based on gene expression. Additionally, both C1 and HC1 showed high gene densities, confirming these trends.

### 3.4 Comparison with Hi-C subcompartments and Segway-GBR domains

We also examined the overlap rates of the domain types with two previously determined genome domain classifications: the Hi-C subcompartments (Rao *et al.* 2014) and the Segway-GBR domain types (Libbrecht *et al.* 2015). Note that the ARIs between the CDACHIE annotations and these classifications are 0.28 and 0.29, respectively, which are slightly smaller than the ARI with HMM_combined in the GM12878 cell line.

Rao *et al.* (2014) introduced a classification of the genome into A/B-compartments (transcriptionally active and inactive regions, respectively) and further subcompartments (A1, A2, B1, B2, B3, and B4) based solely on Hi-C contact matrices. Figure 6b show the overlap rates of the domain types identified by CDACHIE with the Hi-C subcompartments, where the rows and columns are normalized, respectively.

We observe that most of the regions of C1 are equally covered by those of A1 and A2 (Fig. 6b, left). Additionally, 82% of the regions of A1 and 58% of those of A2 are overlapped by C1 (Fig. 6b, right). The features of A1 and A2 described in Rao *et al.* (2014) that are also observed in C1 include:

- high gene density (Fig. 7c),
- high gene expression levels (Fig. 7a),
- high levels of activating chromatin marks, including H3K36me3, H3K79me2, H3K27ac, and H3K4me1 (Fig. 5a).

Half of the regions of C2 are covered by A2. Conversely, the regions of A2 are distributed as follows: 58% overlap with C1, 28% with C2, and 13% with C3. These results suggest that the transcriptionally active domain A2—characterized as less active than A1 based solely on Hi-C data—is further subdivided into C1, C2, and C3 in the classification derived from integrated epigenomic and Hi-C data using CDACHIE. In particular, C2 closely resembles A2 in its histone modification patterns, exhibiting high enrichment in activating chromatin marks, including H3K36me3, H2A.Z, H3K27ac, H3K4me1, H3K4me2, H3K4me3, H3K79me2, and H3K9ac, as well as in the inactivating chromatin mark H3K9me3. Additionally, C2 shows neutral enrichment for the activating chromatin mark H4K20me1 and the inactivating chromatin mark H3K27me3 [compare Fig. 5a and Fig. 2D in Rao *et al.* (2014]. Thus, the integration of epigenomic and Hi-C data by CDACHIE may enhance the resolution of domain A2.

In terms of replication timing, both C1 and A1 show peak enrichment during the G1 phase and remain enriched through the S1 phase [Fig. 5a and Fig. 2D in Rao *et al.* (2014)]. A2 is enriched from the G1 to S2 phases, with a peak in the G1 phase, whereas C2 exhibits a broader enrichment from the G1 to S3 phases, peaking in the S2 phase. These differences indicate that the distinction between C1 and C2 is more pronounced than that between A1 and A2.

C3 corresponds to B1, as 54% of the regions in C3 are covered by B1, and 69% of the regions in B1 are covered by C3 (Fig. 6b). B1 is characterized as facultative heterochromatin due to its positive enrichment in H3K27me3 and negative enrichment in H3K36me3 (Rao *et al.* 2014). This chromatin feature is also observed in C3 (Fig. 5a). In addition, B1 exhibits a peak in replication during the middle of the S phase, and C3 shows a closely similar replication timing pattern (Fig. 5a).

C4 and C5 are associated with B3, as 65% of the regions in C4 and 77% of the regions in C5 are covered by B3, while 36% and 40% of the regions in B3 are covered by C4 and C5, respectively (Fig. 6b). B3 (and B2) has been reported to lack all histone modifications, as shown in Fig. 2D in Rao *et al.* (2014), and exhibits a peak of replication timing during the S4 and G2 phases.

However, C4 shows the second-highest fold enrichment of H3K27me3, an inactive histone modification, which is not observed in B3. Additionally, the peak of replication timing in C4 occurs during the S3 and S4 phases, which is earlier than in B3. These findings suggest that CDACHIE has identified distinct subcategories of inactive regions, with C4 representing a more characteristic inactive domain type than B3.

C6 largely corresponds to B2, with 68% of the regions in C6 being covered by B2, and 45% of the regions in B2 being covered by C6 (Fig. 6b). As mentioned earlier, both B2 and C6 represent less characteristic inactive domain types.

In Rao *et al.* (2014), the B4 subcompartment was reported to span only 11 Mb, accounting for just 0.3% of the genome. This subcompartment is characterized by the coexistence of both active and inactive histone modifications, including H3K36me3 (active) and H3K9me3 and H4K20me3 (inactive). Additionally, its replication timing spans the G1 to S3 phases, with peaks in the S1 and S2 phases, as shown in Fig. 2D of Rao *et al.* (2014).

In the CDACHIE annotation, B4 is included within the C2 domain. This assignment is biologically plausible, as C2 exhibits strong fold enrichments for both H3K36me3 and H3K9me3 (Fig. 5a). (Note that H4K20me3 is not included in the input features of CDACHIE.) Moreover, the replication timing of C2 also spans the G1 to S3 phases, with a peak in the S2 phase—closely corresponding to the timing observed in B4. Therefore, B4 and C2 share highly similar features in terms of both epigenomic marks and replication timing.

In conclusion, the domains inferred by CDACHIE correspond to Hi-C sub-compartments as follows: C1 aligns with A1 and part of A2; C2 spans A2 and B4; C3 matches B1; C4 and C5 map to B3; and C6 corresponds to B2. By jointly leveraging epigenomic and Hi-C data, CDACHIE delineates a richer and more nuanced set of domain categories than Hi-C sub-compartments alone.

We also compared the domains delineated by CDACHIE with those produced by Segway-GBR (Libbrecht *et al.* 2015), a combinatorial annotation framework that couples a hidden Markov model of epigenomic marks with graph-based regularization derived from Hi-C contact maps. Segway-GBR assigns chromatin to five domain categories: specific expression (SPC), broad expression (BRD), facultative heterochromatin (FAC), constitutive heterochromatin (CON), and quiescent (QUI). CDACHIE’s facultative heterochromatin domain C3 aligns with FAC, and its inactive domains C6 and C5 correspond to CON and QUI, respectively, whereas the active domain C1 overlaps both BRD and SPC. Residual discrepancies between the Segway-GBR annotation and other schemes, including that of CDACHIE, may reflect differences in the cell lines analyzed (GM12878 for CDACHIE versus IMR90 for Segway-GBR) as well as in the approaches used to integrate Hi-C and epigenomic data. The overlap patterns are summarized in Fig. 6c, with additional details provided in Supplementary Material 11, available as supplementary data at *Bioinformatics* online.

## 4 Discussion

Shokraneh *et al.* (2023) pointed out that one major challenge in joint modeling of 1D and 3D chromatin organization lies in the spatial resolution gap between functional and structural genomic data. While functional assays such as ChIP-seq provide high-resolution signals (5–100 kb), Hi-C-based annotations of subcompartments are typically limited to coarser resolutions (≥100 kb), especially in inter-chromosomal contexts due to noise and sparsity. This resolution mismatch hinders the integration of 1D and 3D modalities at finer genomic scales. To mitigate this issue, CDACHIE leverages contrastive learning to align the embedding spaces of epigenomic signals and Hi-C interaction patterns at a shared 100 kb bin resolution. By encouraging embeddings from the same genomic bin to be similar across the two modalities, our method allows the Hi-C-based structural representations to inherit fine-grained functional specificity. This strategy effectively bridges the resolution gap in the learned embedding space, enabling integrative modeling at 100 kb resolution even when the original Hi-C data are too sparse at 100 kb. Our results suggest that such cross-modal alignment improves the interpretability and resolution of chromatin domain annotations, addressing a key limitation highlighted in the prior work (Shokraneh *et al.* 2023).

## 5 Conclusions

Our method, CDACHIE, uses two internal encoders to generate embedding vectors separately from Hi-C and epigenetic data within a contrastive learning framework. The embedding vectors corresponding to the same genomic bins are concatenated and subsequently clustered using *K*-means clustering to identify domain types. When evaluated using replication timing, as well as CTCF and RNAP II ChIA-PET loop data, CDACHIE outperforms existing methods, HMM_combined and GMM_GBR. Furthermore, our analysis reveals a relationship between domain annotations and GC dinucleotide sequences, which are known to be associated with gene regulation. These findings suggest that integrating Hi-C and epigenetic data at a higher semantic level via contrastive learning is more effective for capturing genome features than conventional approaches that simply concatenate these data types.

This study has several limitations. First, the domain annotations generated by CDACHIE were evaluated using gene expression, replication timing, CTCF and RNAP II ChIA-PET loop, and dinucleotide sequence data. However, the evaluation was limited to these features, and incorporating additional genomic and epigenomic features could further enhance the analysis in future studies. Second, the evaluation was conducted using data from the GM12878 and K562 cell lines. Expanding the analysis to additional cell lines could provide broader insights into the generalizability of CDACHIE. Finally, CDACHIE represents the first approach to semantically integrate Hi-C and epigenetic data into vector representations. Future studies could explore more sophisticated methods for multi-omics data integration to further improve domain annotation accuracy.

## Supplementary Material

btaf464_Supplementary_Data

## Data Availability

The data underlying this article are available in its online supplementary material.
