# Supplementary material for: CDACHIE: chromatin domain annotation by integrating chromatin interaction and epigenomic data with contrastive learning
Source: Bioinformatics. 2025 Aug 22;41(9):btaf464. doi: 10.1093/bioinformatics/btaf464 (PMC12422559; doi:10.1093/bioinformatics/btaf464)
Supplement: btaf464_Supplementary_Data [file btaf464_supplementary_data.pdf]

Supplementary Data for "CDACHIE: Chromatin Domain Annotation by Integrating Chromatin Interaction and Epigenomic Data with Contrastive Learning"

Asato Yoshinaga* Osamu Maruyama†

Supplementary materials

S1 Data sources

The input data for CDACHIE include a Hi-C contact matrix and various epigenetic datasets for the GM12878 and K562 cell lines. Hi-C data were obtained from the GEO database (accession number GSE63525) [1]. The epigenetic features included DNase I hypersensitive sites (DHS), H2A.Z, and ten histone modifications: H3K4me1, H3K4me2, H3K4me3, H3K9ac, H3K9me3, H3K27ac, H3K27me3, H3K36me3, H3K79me2, and H4K20me1. Supplementary Table S1 summarises these 12 epigenomic marks—ten histone modifications, one histone variant and DNase-I hypersensitivity—and indicates for each whether it is typically associated with transcriptionally *active* or *inactive* chromatin.

To evaluate the inferred annotations, we also incorporated gene expression, replication timing, and CTCF and RNAP II ChIA-PET data following the methodology described in [2]. All datasets were downloaded from Table S1 (Data sources) in [2].

S2 Data preprocessing

The Hi-C data were preprocessed as described in [2]. First, to remove the local bias inherent in Hi-C data, the raw contact matrix was normalized by dividing the observed values by the expected values based on genomic distance to obtain O/E scores at a 100 kb resolution using Juicer [3]. Next, to remove the chromosome bias in the Hi-C data, we created a Hi-C graph by selecting e edges, with the highest O/E scores according to the lengths of chromosomes i and j. e is defined as follows:

$$e = \frac{l_i \times l_j}{(l_{21})^2} \times 2000$$

where l_i and l_j are the lengths of the chromosomes i and j, respectively. We chose 2000 interactions from the intrachromosomal matrix of chromosome 21 (the smallest chromosome).

*Graduate School of Design, Kyushu University

†Faculty of Design, Kyushu University, maruyama.osamu.158@m.kyushu-u.ac.jp

The Hi-C graph was used as the input for LINE (see Supplementary Material S3) to create an embedding vector for each bin.

ChIP-seq data were used to calculate the mean of each epigenomic feature within each of the 100 equally-sized sub-bins (1 kb each) composing a 100 kb bin, using pyBigWig [4]. Each epigenomic feature was standardized using the mean and standard deviation of features across all bins.

The domain annotation generated by CDACHIE was evaluated based on gene expression, replication timing, CTCF and RNAP II [5] ChIA-PET data. The evaluation data and preprocessing procedures are the same as those used by [2].

First, we obtained the RNA-seq in Reads Per Kilobase of transcript per Million mapped reads (RPKM). We retrieved the gene expression values for each domain annotation label across all genes and applied an inverse hyperbolic sine transformation to the gene expression values for reducing the influence of outliers. Subsequently, we calculated the proportion of variance explained by gene expression (GE VE). This metric quantifies the extent to which domain annotation accounts for the gene expression patterns.

Secondly, Repli-seq data were obtained from the ENCODE consortium [6]. For each replication phase, we calculated the proportion of variance explained. We then calculated the average proportion of the variance explained across all six phases to compute the final proportion of variance explained for the replication timing (RT VE).

Third, we obtained the CTCF and RNAP II ChIA-PET data from the 4DN data portal. We lifted over the CTCF and RNAP II bedpe files from hg38 to hg19 using the UCSC liftOver tool. We then calculated the observed over expected ratios for the CTCF and RNAP II ChIA-PET loops (CTCF O/E and RNAP II O/E). This metric quantifies the extent to which domain annotation agrees with the ChIA-PET loops.

S3 LINE, node embedding method

We used LINE to construct the embedding vector of each bin from a Hi-C contact matrix, as described in [7, 2]. LINE assigns embedding vectors to nodes while preserving node proximity in a graph $G = (V, E)$, where V represents bins and E denotes edges that capture their proximity [8]. Two types of proximity are considered: *first-order* proximity and *second-order* proximity. LINE embeddings based on the *second-order* proximity is reported to be more effective for capturing global chromatin structural features compared with those based on *first-order* proximity [2]. Therefore, in this study, we only chose the *second-order* proximity.

The *second-order* proximity is defined as the probability that two nodes have similar neighbors (context nodes) in the graph. The inner product of the embedding vectors $\mathbf{u}_i$ and $\mathbf{u}'_j$ of node v_i and context node v_j is input into the softmax function, which outputs the probability $p_2(v_j|v_i)$, where a connection from node v_i to context node v_j is defined as

$$p_2\left(v_j|v_i\right) = \frac{\exp\left(\mathbf{u}_j'^{\mathrm{T}} \cdot \mathbf{u}_i\right)}{\sum_{k \in V} \exp\left(\mathbf{u}_k'^{\mathrm{T}} \cdot \mathbf{u}_i\right)}.$$

the embedding vectors of the nodes are optimized such that the Kullback-Leibler divergence between $p_2(v_j|v_i)$ and $\hat{p}_2(v_j|v_i) = \frac{w_{ij}}{d_i}$ (d_i is the out-degree of node v_i) is minimized.

We used the original implementation of LINE [8] to construct the embedding vectors of chromosomal segments. We set the dimensions of embedding vectors to 128 and the number of sampled edges during training to 50 million.

S4 Structural and functional encoders

As outlined in Fig. 1, CDACHIE incorporates structural and functional encoders. Raw Hi-C data are processed into a Hi-C contact matrix at a resolution of 100 kb. This matrix is then converted to a graph, where each node represents a 100 kb bin of the chromosome. Graph edges are defined by selecting the top e scored edges, as described in Supplementary Material S2. The resulting graph is input into LINE, which generates 128-dimensional embedding vectors for the bins, referred to as Hi-C embedding vectors. These embedding vectors are subsequently fed into the structural encoder, a ResNet-based deep neural network [9], which outputs 16-dimensional vectors. Details of the architecture are provided in the ”Structural encoder” section. The output vectors are normalized to have a unit norm, and the resulting vectors are referred to as structural embedding vectors.

Next, we describe the functional encoder. Each 100 kb bin of a chromosome is subdivided into 100 equally sized sub-bins. The average signal of each epigenetic marker in each sub-bin forms the epigenetic feature vector for the bin. This feature vector is input into the functional encoder, which is a transformer-based deep neural network [10] that produces a 16-dimensional embedding vector Details of the architecture are provided in the ”Functional encoder” section. The output vector is normalized to have a unit norm and is referred to as the functional embedding vector.

Structural encoder

The structural encoder $\mathrm{E_s}$ is designed to convert a 128-dimensional embedding vector generated by LINE from Hi-C data into a 16-dimensional normalized embedding vector. This encoder employs Tabular ResNet, a variant of ResNet that specializes in tabular data [9]. ResNet is a deep neural network architecture that utilizes skip connections, and is reported to be effective for image tasks [11]. $\mathrm{E_s}$ can be formulated as follows:

$$\mathrm{E_s}(\mathbf{x}) = \mathrm{LastLayer}(\mathrm{ResNetBlock}(\ldots(\mathrm{ResNetBlock}(\mathrm{Linear}_{128,128}(\mathbf{x}))))).$$

The first layer, $\mathrm{Linear}_{128,128}(\mathbf{x})$, converts a 128-dimensional input vector $\mathbf{x}$ into another of the same dimension. The subsequent layer is a stack of four ResNetBlocks. The architecture of ResNetBlock is represented as:

$$\mathrm{ResNetBlock}(\mathbf{x}) = \mathbf{x} + \mathrm{Linear}_{128,128}(\mathrm{GEGLU}(\mathrm{Linear}_{128,256}(\mathrm{BatchNorm}(\mathbf{x})))).$$

The GEGLU (Gated Linear Unit with GELU) activation function [12] applies a Gaussian Error Linear Unit (GELU) activation to the first half of the input vector, whereas the second half remains unchanged by an identity function. The final output is then obtained by performing an element-wise multiplication of these two halves. The final operation of $\mathrm{E_s}$, denoted by LastLayer, executes LayerNorm, ReLU, $\mathrm{Linear}_{128,64}$, and $\mathrm{Linear}_{64,16}$, sequentially:

$$\mathrm{LastLayer}(\mathbf{x}) = \mathrm{Linear}_{64,16}(\mathrm{Linear}_{128,64}(\mathrm{ReLU}(\mathrm{LayerNorm}(\mathbf{x})))).$$

Functional encoder

The functional encoder, denoted by $\mathrm{E_f}$, converts an input epigenomic feature matrix into a 16-dimensional normalized embedding vector. The input matrix, constructed from 12 epigenomic features, is represented as $X = (x_1, \ldots, x_{100})^{\mathrm{T}} \in \mathbb{R}^{100\times 12}$. The ith row is a 12-dimensional vector, whose jth element is the mean of the jth epigenetic signal over the ith 1 kb-sub-bin

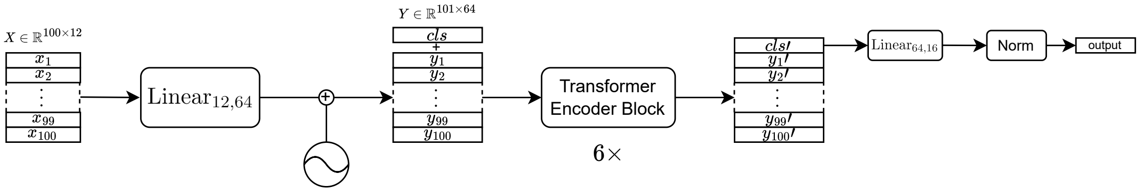

(a)

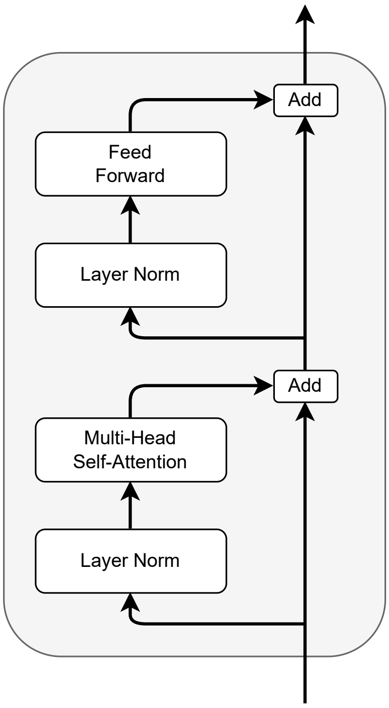

(b)

Fig. S1: (a) Structure of the Functional Encoder inputs an epigenomic feature matrix $X \in \mathbb{R}^{100\times 12}$ and outputs a 16-dimensional normalized embedding vector. (b) The architecture of the TransformerEncoderBlock includes multi-head attention and a Feed Forward Network.

within a 100 kb bin. The architecture of $\mathrm{E_f}$ is given by: Fig. S1 (a). The network first applies a fully connected layer $\mathrm{Linear}_{12,64}$ to the input matrix X. The resulting matrix is then passed to the PositionalEncoder function that adds positional encoding vectors to each vector in the matrix. Next, a CLS vector representing the embedding of the entire input is added at the beginning of the matrix. The resulting matrix $Y \in \mathbb{R}^{101\times 64}$ is then passed on to a stack of six basic Transformer Encoder blocks TransformerEncoderBlock. Finally, $\mathrm{E_f}$ applies a fully connected layer, $\mathrm{Linear}_{64,16}$, to the CLS vector.

Here, we describe the architecture of TransformerEncoderBlock depicted in Fig. S1 (b) including a multihead attention layer. Multihead attention is computed from Y as follows:

$$\mathrm{MultiHead}(Y) = \mathrm{concat}(\mathrm{head}_1, \mathrm{head}_2, \ldots, \mathrm{head}_h)W^O$$

where

$$\mathrm{head}_i = \mathrm{Attention}(YW_i^Q, YW_i^K, YW_i^V),$$
$$\mathrm{Attention}(Q, K, V) = \mathrm{softmax}\left(\frac{QK^{\mathrm{T}}}{\sqrt{d_k}}\right)V, \text{ and}$$
$$d_k = \frac{d_{model}}{h}$$

Note that W_i^Q, W_i^K, W_i^V, W^O are learnable weight matrices. Function concat concatenates the given arguments. Function Attention calculates the dot products of Q and K^{T}, divides the result by $\sqrt{d_k}$, and applies the softmax function. The result represents normalized similarity (weight) measures from every query to the key and value. Finally, each query vector is updated using the weighted sum of the value vectors V. d_{model} is the dimension of the vector of the input matrix Y. h denotes the number of heads. In this study, $h = 8$ and $d_{model} = 64$. The FFN is defined as follows:

$$\mathrm{FFN}(y) = \mathrm{Linear}_{d_{ff}, d_{model}}(\mathrm{GEGLU}(\mathrm{Linear}_{d_{model}, 2d_{ff}}(y)))$$

where y is each vector of the input matrix Y, and d_{ff} is the dimension of the hidden layer in FFN. In this study, we set $d_{ff} = 256$.

S5 Contrastive learning

The contrastive learning process trains structural and functional encoders to maximize the cosine similarity between the functional and structural vectors of the same bins while minimizing the cosine similarity between the vectors of different bins (see Fig. 1). The contrastive loss function, L, is formulated as follows: A minibatch of size n is formed by randomly sampling n bins from the dataset. The cross-entropy loss function denoted by L_{CE} is applied to two sequences of embedding vectors from the minibatch, $\mathbf{X} = \{\mathbf{x}_1, \ldots, \mathbf{x}_n\}$ and $\mathbf{Y} = \{\mathbf{y}_1, \ldots, \mathbf{y}_n\}$ is defined as follows:

$$L_{CE}(\mathbf{X}, \mathbf{Y}) = -\frac{1}{n}\sum_{i=1}^{n}\sum_{j=1}^{n}\delta_{ij}\log\frac{\exp(\mathrm{sim}(\mathbf{x}_i, \mathbf{y}_j)/T)}{\sum_{k=1}^{n}\exp(\mathrm{sim}(\mathbf{x}_i, \mathbf{y}_k)/T)}$$

where δ_{ij} is the Kronecker delta function, which equals 1 if $i = j$ and 0 otherwise; $\mathrm{sim}(\mathbf{x}_i, \mathbf{y}_j)$ represents the cosine similarity between $\mathbf{x}_i$ and $\mathbf{y}_j$; and where T is the temperature parameter. Notably, the argument in the log term corresponds to the output of the softmax function, normalized across the second sequence of embedding vectors $\mathbf{Y}$. Let the functional and structural

embedding vectors be denoted as $\mathbf{F} = (\mathbf{f}_1, \mathbf{f}_2, \ldots, \mathbf{f}_n)$ and $\mathbf{S} = (\mathbf{s}_1, \mathbf{s}_2, \ldots, \mathbf{s}_n)$, respectively. The contrastive loss function L for $\mathbf{F}$ and $\mathbf{S}$ is defined as the average of $L_{CE}(\mathbf{S}, \mathbf{F})$ and $L_{CE}(\mathbf{F}, \mathbf{S})$:

$$L = \frac{1}{2}(L_{CE}(\mathbf{S}, \mathbf{F}) + L_{CE}(\mathbf{F}, \mathbf{S})).$$

The hyperparameters used to train the contrastive learning encoders are listed in Supplementary Table S2.

S6 Evaluation metrics

The variance explained (VE) and observed-to-expected ratio (O/E) are employed to assess the quality of domain annotations.

Proportion of variance explained The VE quantifies the consistency of a specific feature within each domain type of a domain annotation [2]. The VE of a feature over an annotation is defined as

$$\mathrm{VE} = \frac{\mathrm{var}\left(\{s_i\}\right) - \mathrm{var}(\{s_i - \mu_i\})}{\mathrm{var}(\{s_i\})} = 1 - \frac{\sum_{i=1}^{n}(s_i - \mu_i)^2}{\sum_{i=1}^{n}(s_i - \mu)^2}$$

where s_i is the feature value in the i-th bin, μ_i denotes the mean feature values of bins sharing the same domain type as the i-th bin, μ is the mean feature value across all bins, and n is the number of bins. VE ranges from 0 to 1, with higher scores indicating better agreement between the annotation and feature.

ChIA-PET loop analysis of domain annotations using O/E We evaluate whether the two ends of the CTCF and RNAP II ChIA-PET loops belong to the same domain type using the O/E ratio framework, as described in [2]. The O/E ratio is a statistical measure that compares the observed frequency of a feature to its expected frequency. For $i \leq j$, let $O_{i,j}$ be the number of observed loops between an end belonging to the i-th domain type and the other end belonging to the j-th domain type. Let C_i be the coverage of the i-th domain type. The expected number of loops between the i-th and j-th domain types is $E_{i,j} = T \cdot C_i \cdot C_j$ where $T = \sum_{i=1}^{K} \sum_{j=i}^{K} O_{i,j}$ is the total number of loops and K is the number of domain types. The O/E is defined as follows:

$$\mathrm{O/E} = \sum_{i=1}^{K} \frac{O_{i,i}}{E_{i,i}}.$$

The O/E ranges from 0 to ∞, and a higher O/E score indicates stronger agreement with the ChIA-PET loop structures.

S7 Evaluation of cluster number, K

We evaluated the performance of CDACHIE across a range of cluster numbers from $K = 6$ to $K = 10$. The Silhouette coefficient measures how similar a sample is to its own cluster compared to other clusters; it ranges from -1 to 1, with higher values indicating better clustering quality. In contrast, the Davies-Bouldin index quantifies the average similarity between each cluster and its most similar counterpart; it ranges from 0 to ∞, with lower values indicating better

clustering quality. As shown in Supplementary Fig. S2 (a), both the Silhouette coefficient and the Davies-Bouldin index remain relatively consistent across this range. This consistency suggests that increasing K beyond 6 does not substantially improve clustering quality in terms of compactness or separation.

In Fig. S2 (b), we observe that the variance explained for both replication timing (RT VE) and gene expression (GE VE) remains relatively consistent across different values of K. In contrast, the observed/expected (O/E) ratios for CTCF and RNAPII increase approximately linearly with K. However, at $K = 10$, the fold enrichment patterns become less distinct (Supplementary Fig. S2 (c)), which is consistent with previous findings [2] suggesting that the interpretability of domain annotations tends to decline as K increases. Therefore, we selected $K = 6$ for our main analysis to ensure both comparability with prior studies and clear interpretability of the results.

S8 Adjusted rand index

The ARI adjusts the Rand Index (RI), defined as follows: Let $X = \{X_1, X_2, \ldots, X_r\}$ and $Y = \{Y_1, Y_2, \ldots, Y_s\}$ be two partitions for a set of n elements. The RI is defined as

$$\mathrm{RI} = \frac{a + b}{\binom{n}{2}}$$

where a is the number of element pairs in the same subset in both X and Y, and b is the number of element pairs in different subsets in both X and Y. The ARI is defined as

$$\mathrm{ARI} = \frac{\mathrm{RI} - E[\mathrm{RI}]}{\max(\mathrm{RI}) - E[\mathrm{RI}]}$$

where $E[\mathrm{RI}]$ is the expected value of RI for random clusterings, and max(RI) is the maximum possible RI value.

S9 Embedding vector distribution visualized using UMAP

To further investigate the functional features of domain annotations, we visualized 2D projections of the concatenated embedding vectors for epigenomic and Hi-C data using Uniform Manifold Approximation and Projection (UMAP). Fig. S3 (a) and (b) show the domain annotations of CDACHIE and HMM_combined, respectively, colored according to domain type. The domain types identified by CDACHIE demonstrate a clear separation, reflecting the sensitivity of the method to structural and functional differences.

Fig. S3 (c)-(f) illustrate the enrichment of H3K27ac (an activating histone modification) and H3K27me3 (a repressive histone modification). In CDACHIE (Fig. S3 (c)), regions with high H3K27ac enrichment primarily coincide with the active domain types C1 and C2. In contrast, HMM_combined (Fig. S3 (d)) shows a broader distribution of H3K27ac across HC1 through HC3. A similar pattern is observed for H3K27me3 (Fig. S3 (e) and (f)), with regions of high enrichment aligning with inactive domain types in both methods: C3-C4 in CDACHIE and HC4 in HMM_combined. These findings are consistent with the fold enrichment results presented in Fig. 5. The projections of the remaining histone modifications are given in Supplementary Fig. S4.

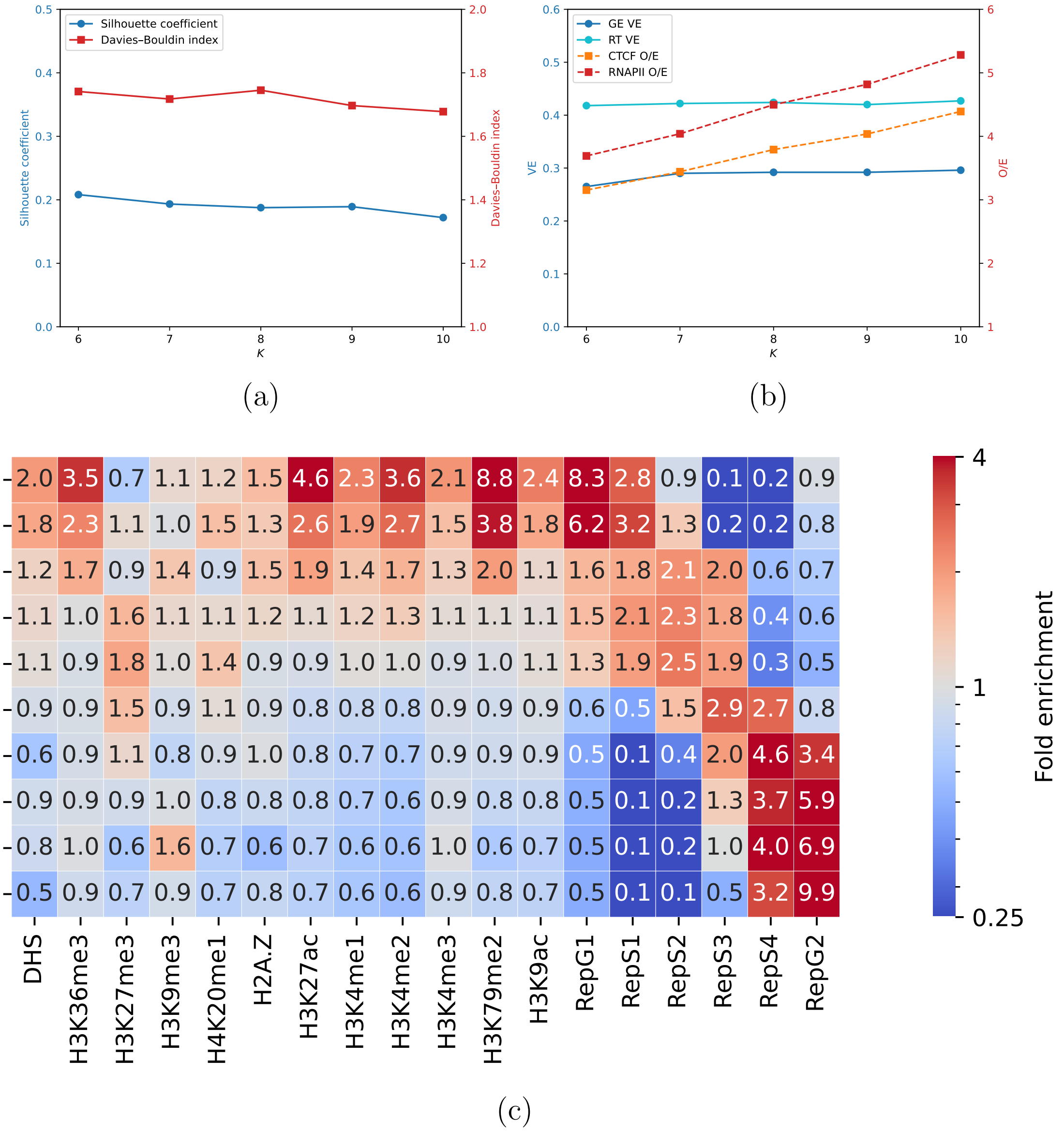

Fig. S2: Analysis of the cluster number, K, ranging from 6 to 10. (a) shows the Silhouette coefficient and Davies-Bouldin index. (b) presents the variance explained for gene expression (GE VE) and replication timing (RT VE), as well as observed-to-expected ratio for CTCF ChIA-PET loops (CTCF O/E) and RNAP II ChIA-PET loops (RNAP II O/E). (c) displays the fold enrichment of epigenomic markers and replication timing signals for each domain type identified by CDACHIE with $K = 10$. Fold enrichment is calculated as the ratio of the median signal value within each domain type to the median value across all genomic bins.

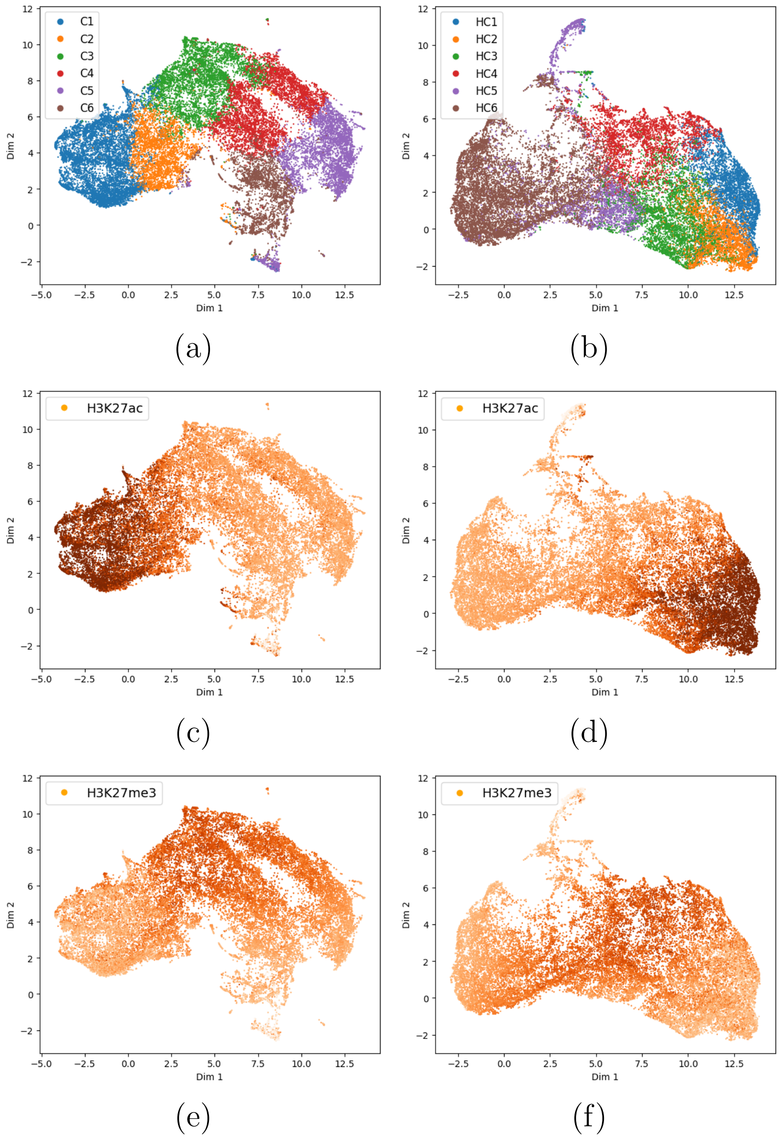

Fig. S3: UMAP 2D projection of concatenated structural and functional embedding vectors from CDACHIE (left) and concatenated epigenomic and Hi-C embedding vectors from HMM_combined (right). Plots are colored based on domain types from CDACHIE (a) and HMM_combined (b), enrichment of H3K27ac in CDACHIE (c) and HMM_combined (d), and enrichment of H3K27me3 in CDACHIE (e) and HMM_combined (f). The distributions of the remaining epigenetic signals over the UMAP 2D projection of concatenated structural and functional embeddings, (a) and (b), are shown in Supplementary Fig. S4.

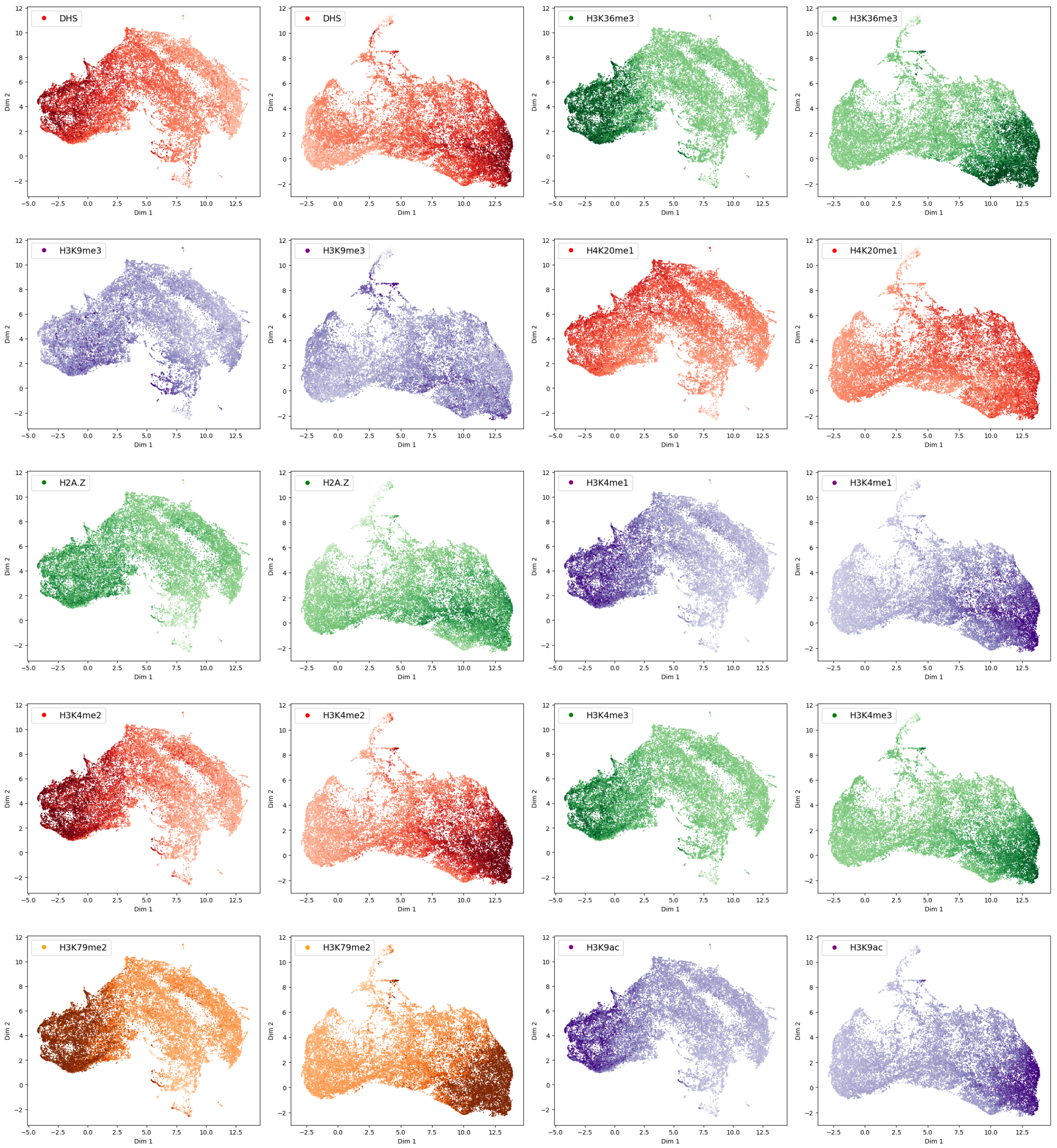

Fig. S4: Epigenetic signal distribution over the UMAP 2D projection of concatenated structural and functional embeddings. For each histone modification, the left and right panels show the results from CDACHIE and HMM_combined, respectively.

S10 Dinucleotide distribution patterns across chromatin domain types

The relationship between CpG distribution and chromatin structure across multiple length scales was investigated [13]. Building on this, we examined the distribution of dinucleotide sequences within the domain types identified using CDACHIE and HMM_combined.

We calculated the enrichment value of a dinucleotide sequence within a given bin, which was defined as the frequency of dinucleotides in that bin divided by the median frequency across all bins in the entire genome. To visualize this, we overlaid Fig. S3 (a) and (b) with colors representing the dinucleotide enrichment. The results for the CpG dinucleotide are shown in the CG panels of Fig. S5. Regions enriched in CpG dinucleotides are often found in promoter regions, such as CpG islands, and are, therefore, associated with gene regulation. In the CDACHIE CG panel, the CpG-enriched regions of CDACHIE partially overlapped with domains C1 and C3, as shown in Fig. S3 (a). The most highly enriched CpG region in the CDACHIE CG panel is located at the upper-left border of the domains C1 and C3. This observation aligns with the gene expression levels and gene densities of C1 and C3. Specifically, C1 and C3 are the two domains with the highest and second highest gene densities, respectively, as shown in Fig. 7 (c). Furthermore, C1 has the highest gene expression levels, whereas C3 had the lowest, as depicted in Fig. 7 (a).

In contrast, within the HMM_combined annotation, we observed that the most CpG-enriched region in the right-hand CG panel of Fig. S5 (HMM combined) lies along the right-middle border, aligning with sections of HC1 and HC4 (see Fig. S3 (b)). HC1 is characterized by high gene density and elevated expression levels (see Fig. 7 (b) and (d)), whereas HC4 exhibits comparatively low gene density and expression.

The overlap relationship between the domain annotations of CDACHIE and HMM_combined is shown in Fig. 6 (a).The domain type that most overlaps with C1 in HMM_combined is HC1, and vice versa. Similarly, the domain type that most overlaps with C3 in HMM_combined is HC4, and vice versa. The CpG enrichment index serves as a key measure in domain annotations because the CpG-enriched regions are conserved across the two domain types in both chromatin domain annotations.

Fig. S5 shows the dinucleotide sequence distribution over the UMAP 2D projection of the concatenated structural and functional embeddings, and Fig. S6 illustrates the fold enrichment of all dinucleotides. We observed that dinucleotides comprising G and C, such as CpC(GpG) and GpC, exhibited similar patterns in both UMAP projections and fold enrichment as CpG. Additionally, regions C4-C6 were found to be enriched in A+T content. This GC content enrichment is consistent with the concept of 'isochore' regions [14, 15, 16], which are relatively large DNA segments in eukaryotic genomes characterized by uniform GC content. Isochores serve as structural chromatin units in the genome. Furthermore, isochore regions have been reported to be correlated with gene density and replication timing [17, 18]. Therefore, the domains identified by CDACHIE and HMM_combined show a significant relationship with isochore regions.

S11 Comparison with Segway-GBR domains

Fig. 6 (c) shows the overlap between the domain types identified by CDACHIE and those from Segway-GBR. Among these, C1 in CDACHIE exhibits substantial correspondence with both SPC and BRD: 37% and 47% of the C1 regions overlap with SPC and BRD domains,

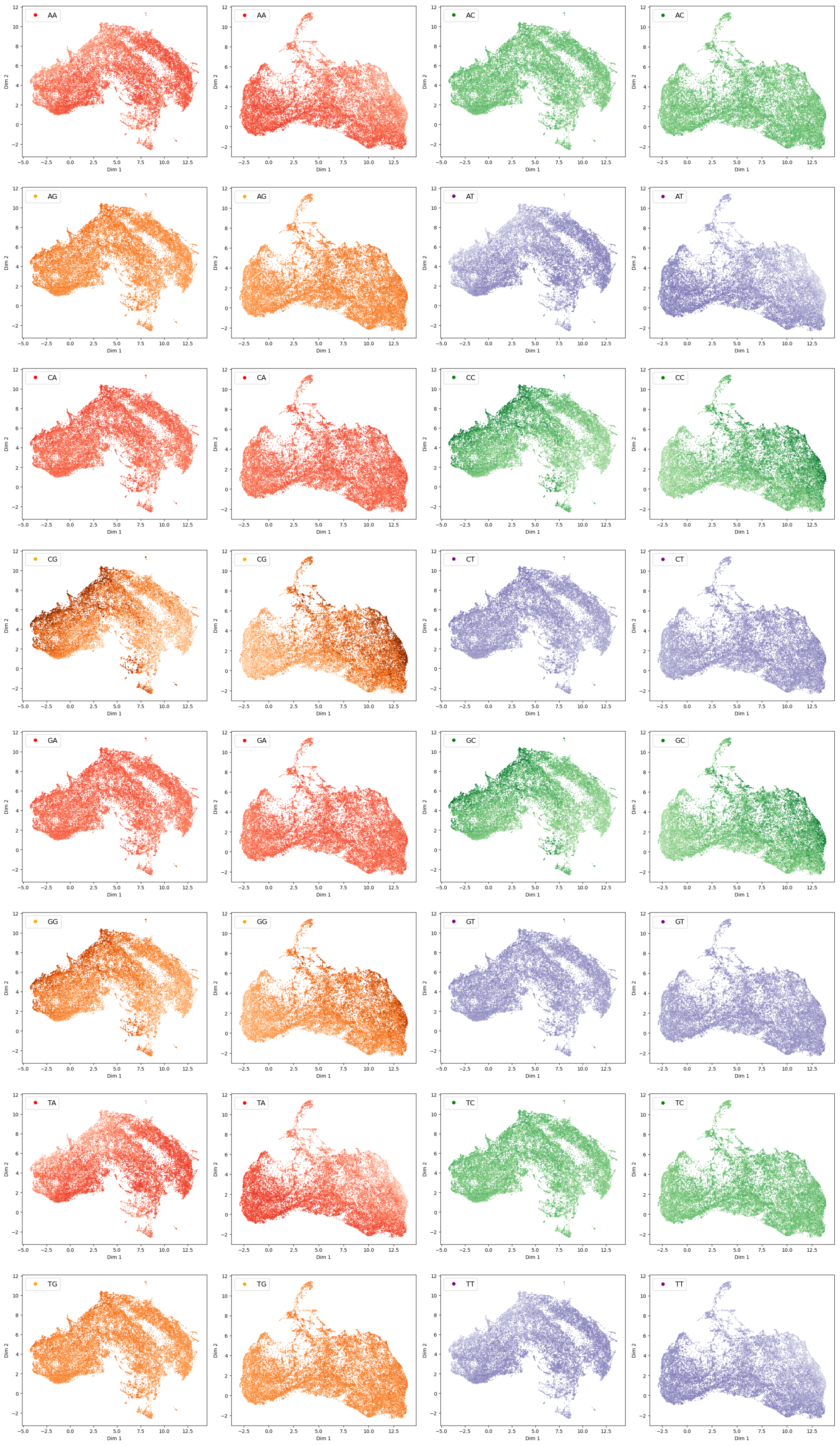

Fig. S5: Dinucleotide sequence distribution over the UMAP 2D projection of concatenated structural and functional embeddings. For each dinucleotide sequence, the left and right panels show the results from CDACHIE and HMM_combined, respectively.

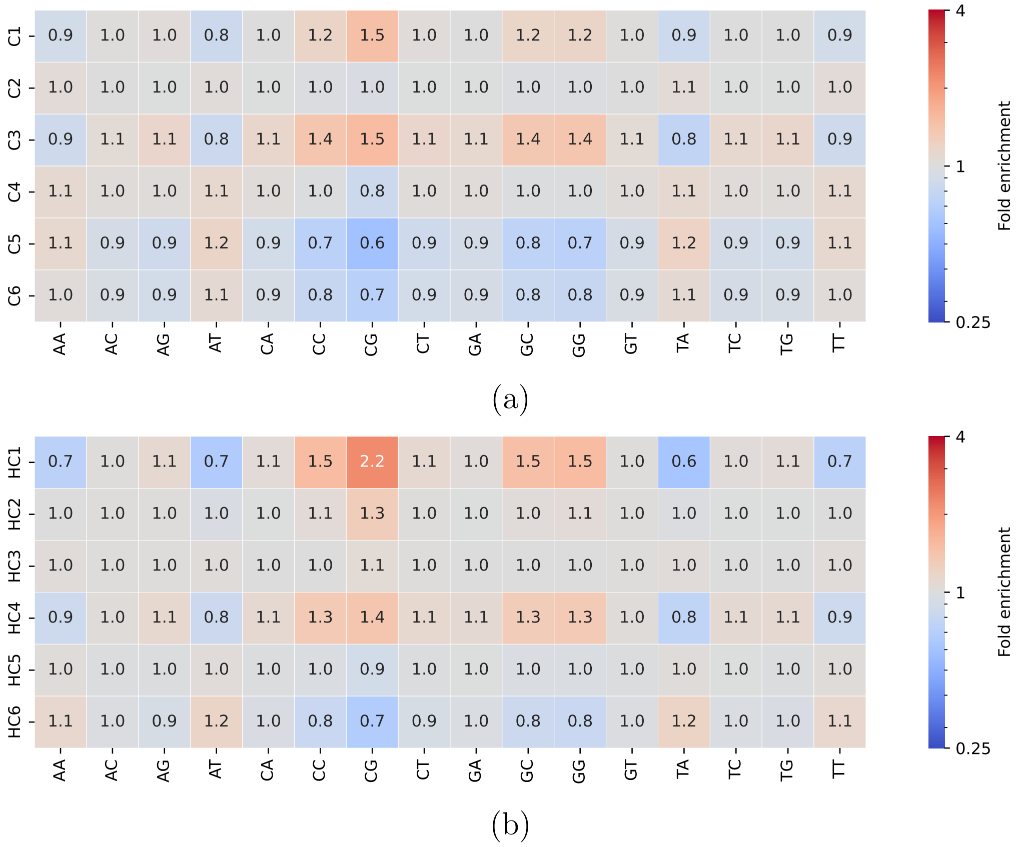

Fig. S6: Fold enrichment of the dinucleotide sequence of each domain type from the CDACHIE (a) and HMM_combined (b) domain annotations.

respectively, while 89% of SPC and 70% of BRD regions are contained within C1. In other words, the majority of SPC and BRD regions are included within C1.

SPC is characterized by high enrichment of all histone modifications, except for the inactivating chromatin marks H3K27me3 and H3K9me3, which are low (Fig. 2 (A) in [19]). This pattern is also observed in C1 (Fig. 5 (a)), HC1 (Fig. 5 (b)), and B1 (Fig. 2 (D) in [1]).

In contrast, BRD is characterized by high enrichment of H4K20me1 and H3K36me3, with low enrichment of H3K27me3 and H3K9me3. While no domain type in CDACHIE, HMM_combined, or Hi-C subcompartments shows a highly similar histone modification pattern to BRD, the most similar patterns are observed in C1, HC1, and A1, respectively.

C3 and FAC coincide with each other, as 91% of the regions of C3 are covered by FAC, while 42% of the regions of FAC are covered by C3. The characteristics of FAC are high enrichment of H3K27me3, high GC content, relatively high gene density, but low gene expression levels in [19]. These features are also observed in C3, as shown in Fig. 5 (a), Fig. 7 (g), and Fig. 8 (c) and (a), respectively.

CON is primarily represented in C6, with 70% of CON's regions overlapping with C6, as shown in Fig. 6 (c) right. In addition, [19] reported that the replication timing of CON is late, which aligns with the late replication timing observed for C6, S4, and G2 (Fig. 5 (a)).

However, although C6 covers the largest portion of CON's regions, it accounts for only 23% of them. Therefore, there is no evident primary correspondence between CON and the domain types identified by CDACHIE (Fig. 6 (c) left). CON also exhibits similar characteristics to the domain types identified by HMM_combined (Fig. S7 (c)). H3K9me3, an inactive histone modification, is enriched only in CON in the domain annotations of Segway-GBR [19], and the other active and inactive histone modifications are not enriched in CON [19]. However, H3K9me3 is highly enriched in multiple domain types across each of the domain annotations of CDACHIE, HMM_combined, and Hi-C subcompartments: C2 and C6 (Fig. 5 (a)), HC2, HC3, and HC5 (Fig. 5 (b)), and B4 and A2 (Fig. 2 (D) in [1]), respectively. Thus, CON appears to represent a distinct domain type that is not specifically captured by the other methods.

C5 and QUI are largely overlapping, as 93% of the regions of C5 are covered by QUI, while 50% of the regions of QUI are covered by C5. QUI was characterized to be a low gene density, gene expression level, and low enrichment of all histone modifications, which are also observed in C5 (Fig. 8 (c) and (a), and Fig. 5 (a)).

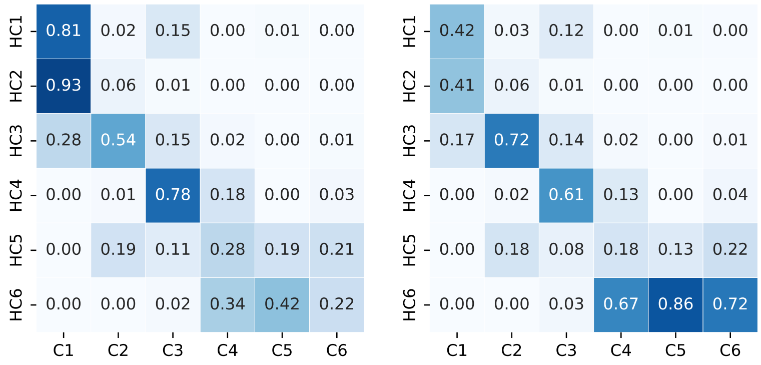

(a) CDACHIE

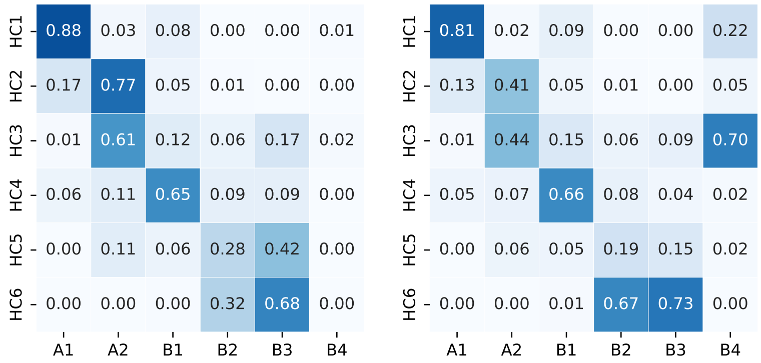

(b) Hi-C subcompartment

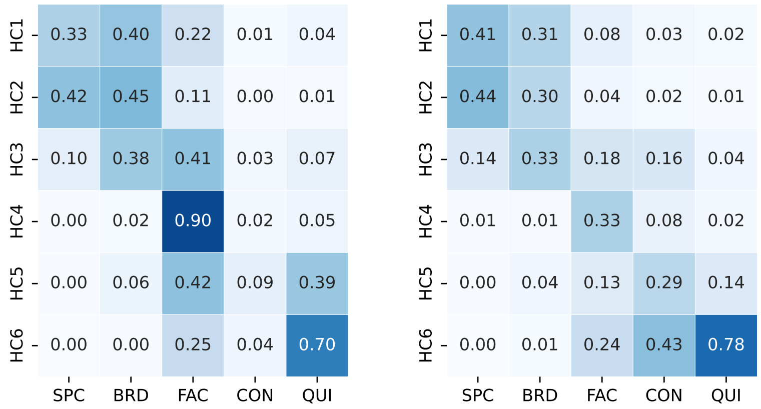

(c) Segway-GBR

Fig. S7: Overlap rates between the domain types identified by HMM_combined and (a) those derived from CDACHIE, (b) Hi-C subcompartments, and (c) those from Segway-GBR for the GM12878 cell line. The left panels show row-normalized values, while the right panels show column-normalized values, respectively.

Supplementary Figures

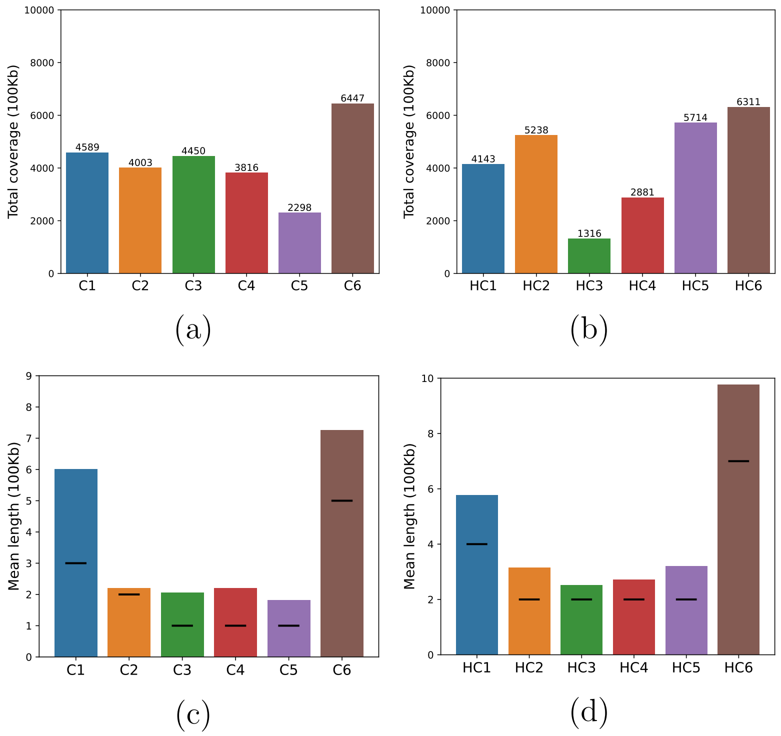

Fig. S8: Number of bins for each domain type (domain coverage) in the domain annotations of CDACHIE (a) and HMM_combined (b) and the mean length of contiguous bins with the same domain type for CDACHIE (c) and HMM_combined (d) for the K562 cell line. The horizontal lines within the bars represent the median. The output of GMM_GBR is not included because the annotation files for the K562 cell line were not available in the reference study [2].

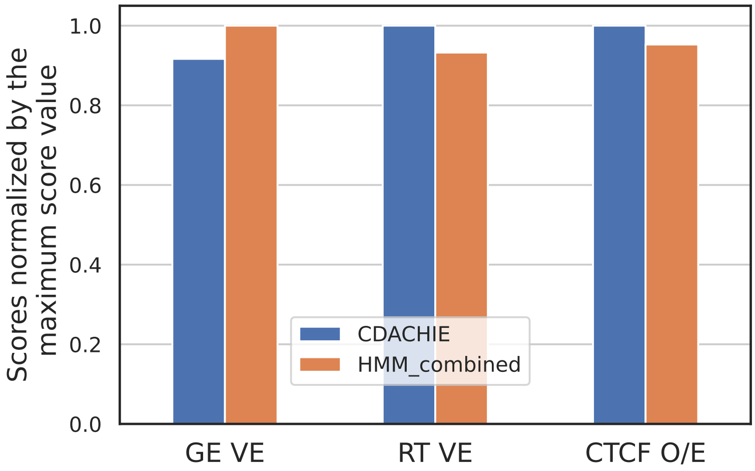

Fig. S9: VE and O/E scores of domain annotations generated using CDACHIE and HMM_combined For the K562 cell line (The experimental data of GMM_GBR were not provided in [2]). The four metrics GE VE, RT VE, and CTCF O/E are shown for each of the tools. The y-axis shows the scores normalized by the maximum score of each metric.

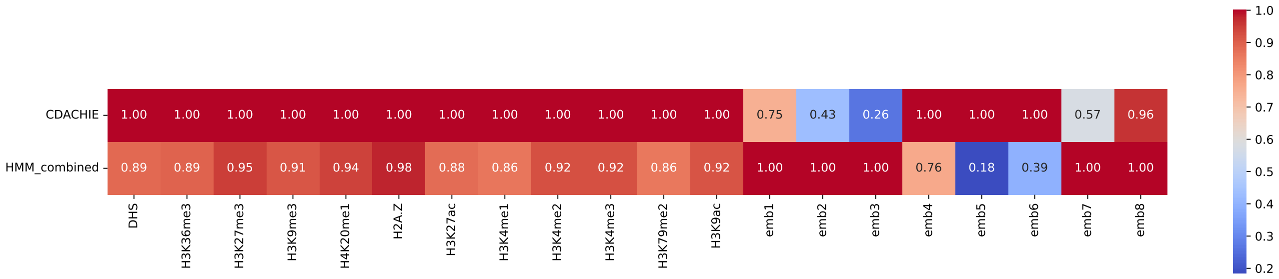

Fig. S10: The normalized proportion of variance explained (VE) for each input signal in the domain annotations of CDACHIE, HMM_combined for the K562 cell line. Each VE is normalized by dividing by the maximum VE across the two annotations, representing the proportions for 12 epigenomic signals and each component of the eight-dimensional Hi-C embedding vectors generated using LINE. The dimensionality of the Hi-C embedding vector is set to eight to match the HMM_combined.

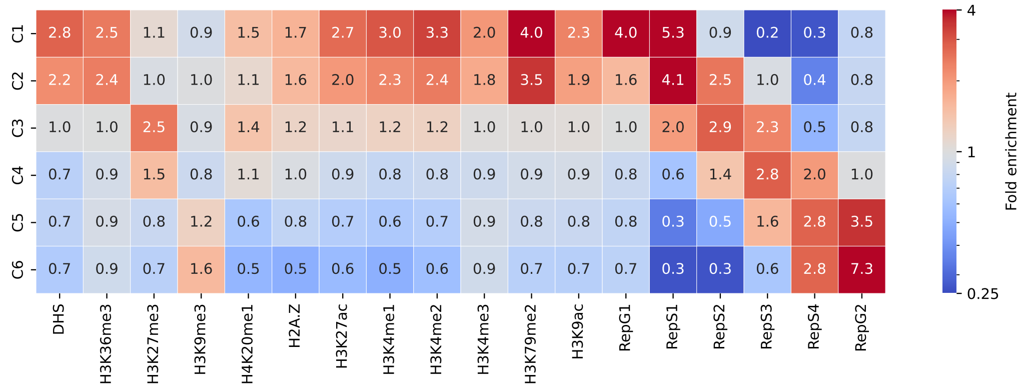

(a)

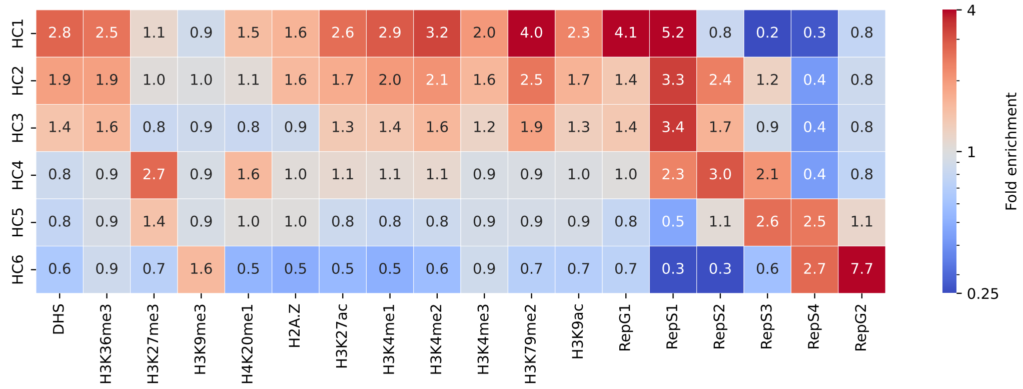

(b)

Fig. S11: Fold enrichment of the epigenomic and replication timing signals of each domain type from CDACHIE (a) and HMM_combined (b) domain annotations for the K562 cell line.

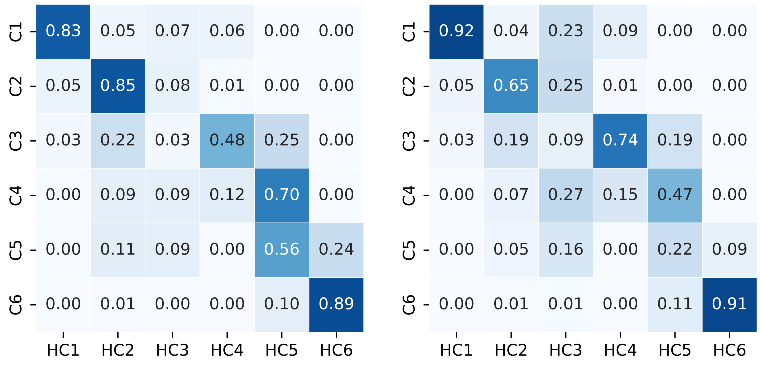

(a) HMM_combined

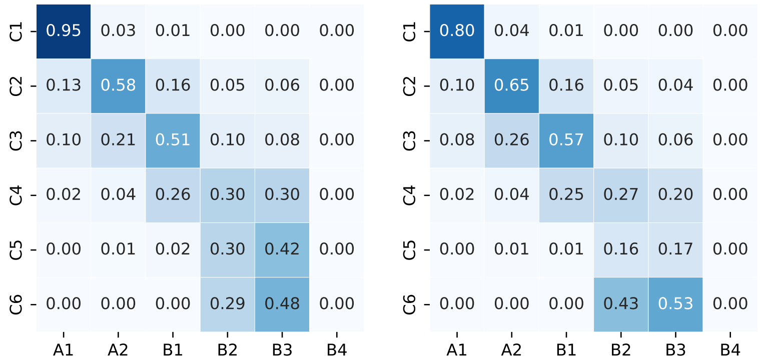

(b) Hi-C subcompartment

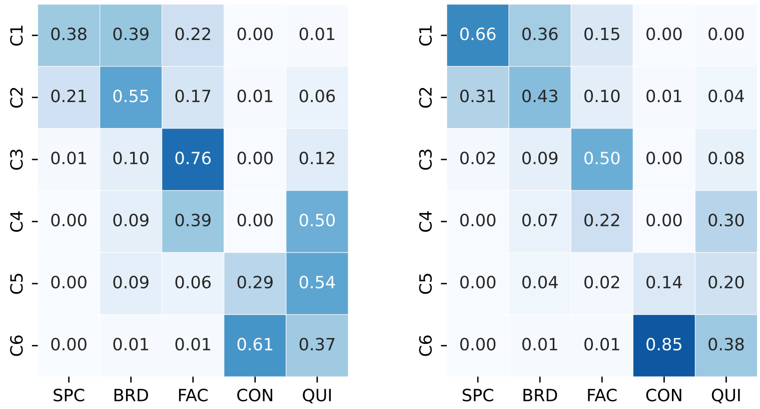

(c) Segway-GBR

Fig. S12: Overlap rates between the domain types identified by CDACHIE and (a) those derived from HMM_combined, (b) Hi-C subcompartments, and (c) those from Segway-GBR for the K562 cell line. The left panels show row-normalized values, while the right panels show column-normalized values, respectively.

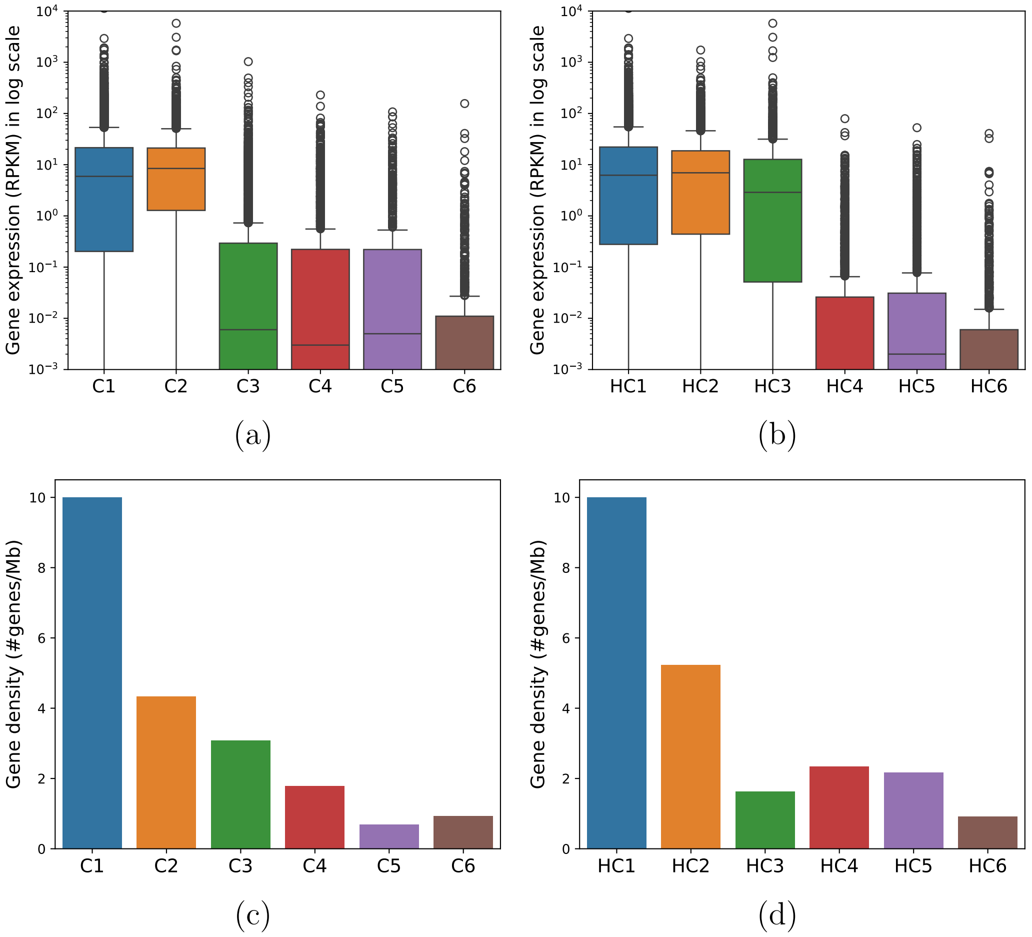

Fig. S13: Distribution of gene expression values in RPKM for each domain type of CDACHIE (a) and HMM_combined (b) domain annotations for the K562 cell line. Gene density corresponding to each domain type of CDACHIE (c) and HMM_combined (d) domain annotations for the K562 cell line.

Supplementary Tables

Table S1: Histone modifications, variants, and chromatin accessibility markers with associated functions and assay methods [20].

Category	Marker	Function
histone modification (ChIP-seq)	H3K36me3	active
	H4K20me1	active
	H3K27ac	active
	H3K4me1	active
	H3K4me2	active
	H3K4me3	active
	H3K79me2	active
	H3K9ac	active
	H3K27me3	inactive
	H3K9me3	inactive
histone variant (ChIP-seq)	H2A.Z	active
chromatin accessibility (DNase-seq)	DNase I hypersensitive sites (DHS)	active

Table S2: Hyperparameters used in the contrastive learning process.

Hyperparameter	Value
learning rate	10^{-5}
batch size	512
epochs	4
temperature (T)	0.5
optimizer	Adam

References

[1] Suhas S. P. Rao, Miriam H. Huntley, Neva C. Durand, et al. A 3D map of the human genome at kilobase resolution reveals principles of chromatin looping. *Cell*, 159:1665–1680, 2014.

[2] Neda Shokraneh, Mariam Arab, and Maxwell Libbrecht. Integrative chromatin domain annotation through graph embedding of Hi-C data. *Bioinformatics*, 39:btac813, 2022.

[3] Neva C. Durand, Muhammad S. Shamim, Ido Machol, et al. Juicer provides a one-click system for analyzing loop-resolution Hi-C experiments. *Cell systems*, 3:95–98, 2016.

[4] pybigwig. `https://github.com/deeptools/pyBigWig/`.

[5] Nastaran Heidari, Douglas H. Phanstiel, Chao He, et al. Genome-wide map of regulatory interactions in the human genome. *Genome research*, 24:1905–1917, 2014.

[6] ENCODE Project Consortium et al. An integrated encyclopedia of dna elements in the human genome. *Nature*, 489:57, 2012.

[7] Haitham Ashoor, Xiaowen Chen, Wojciech Rosikiewicz, et al. Graph embedding and unsupervised learning predict genomic sub-compartments from HiC chromatin interaction data. *Nature communications*, 11:1173, 2020.

[8] Jian Tang, Meng Qu, Mingzhe Wang, et al. Line: Large-scale information network embedding. pages 1067–1077, 2015.

[9] Yury Gorishniy, Ivan Rubachev, Valentin Khrulkov, et al. Revisiting deep learning models for tabular data. volume 34, pages 18932–18943, 2021.

[10] Ashish Vaswani, Noam Shazeer, Niki Parmar, et al. Attention is all you need. *Advances in neural information processing systems*, 30, 2017.

[11] Kaiming He, Xiangyu Zhang, Shaoqing Ren, et al. Deep residual learning for image recognition. pages 770–778, 2016.

[12] Noam Shazeer. Glu variants improve transformer. *arXiv preprint arXiv:2002.05202*, 2020.

[13] Zhicheng Cai, Yueying He, Sirui Liu, et al. Hierarchical dinucleotide distribution in genome along evolution and its effect on chromatin packing. *Life Science Alliance*, 4, 2021.

[14] Giorgio Bernardi, Birgitta Olofsson, Jan Filipski, et al. The mosaic genome of warm-blooded vertebrates. *Science*, 228:953–958, 1985.

[15] Giorgio Bernardi. The vertebrate genome: isochores and evolution. *Molecular biology and evolution*, 10:186–204, 1993.

[16] Giorgio Bernardi. Isochores and the evolutionary genomics of vertebrates. *Gene*, 241:3–17, 2000.

[17] Maria Costantini, Oliver Clay, Fabio Auletta, et al. An isochore map of human chromosomes. *Genome research*, 16:536–541, 2006.

[18] Maria Costantini and Héctor Musto. The isochores as a fundamental level of genome structure and organization: a general overview. *Journal of molecular evolution*, 84:93–103, 2017.

[19] Maxwell W. Libbrecht, Ferhat Ay, Michael M. Hoffman, et al. Joint annotation of chromatin state and chromatin conformation reveals relationships among domain types and identifies domains of cell-type-specific expression. *Genome research*, 25:544–557, 2015.

[20] Zhibin Wang, Chongzhi Zang, Jeffrey A. Rosenfeld, et al. Combinatorial patterns of histone acetylations and methylations in the human genome. *Nature Genetics*, 40:897–903, 2008.
